# Rhizosheath–root system changes exopolysaccharide content but stabilizes bacterial community across contrasting seasons in a desert environment

**DOI:** 10.1186/s40793-022-00407-3

**Published:** 2022-04-01

**Authors:** Ramona Marasco, Marco Fusi, Maria Mosqueira, Jenny Marie Booth, Federico Rossi, Massimiliano Cardinale, Grégoire Michoud, Eleonora Rolli, Gianmarco Mugnai, Lorenzo Vergani, Sara Borin, Roberto De Philippis, Ameur Cherif, Daniele Daffonchio

**Affiliations:** 1 Biological and Environmental Sciences and Engineering Division (BESE), King Abdullah University of Science and Technology (KAUST), Thuwal, Kingdom of Saudi Arabia; 2 Department of Agriculture, Food, Environment and Forestry (DAGRI), University of Florence, Florence, Italy; 3 Department of Biological and Environmental Sciences and Technologies, University of Salento, Lecce, Italy; 4 Institute of Applied Microbiology, Research Center for BioSystems, Land Use, and Nutrition (IFZ), Justus-Liebig-University, Giessen, Germany; 5 Department of Food, Environmental and Nutritional Sciences (DeFENS), University of Milano, Milan, Italy; 6 Institute of BioEconomy, CNR, Sesto Fiorentino, Florence, Italy; 7 Institut Supérieur de Biotechnologie Sidi Thabet (ISBST), BVBGR-LR11ES31, Biotechpole Sidi Thabet, University Manouba, Ariana, Tunisia; 8 Present Address: Joint Nature Conservation Committee, Monkstone House, City Road, Peterborough, PE1 1JY UK

**Keywords:** Rhizosheath, Plant-microbiome, Desert, Extracellular polymeric substances (EPS), Plant legacy, Environmental fluctuation, PGP microorganisms, Desertification, Environmentally-independent microbiome

## Abstract

**Background:**

In hot deserts daily/seasonal fluctuations pose great challenges to the resident organisms. However, these extreme ecosystems host unique microenvironments, such as the rhizosheath–root system of desert speargrasses in which biological activities and interactions are facilitated by milder conditions and reduced fluctuations. Here, we examined the bacterial microbiota associated with this structure and its surrounding sand in the desert speargrass *Stipagrostis pungens* under the contrasting environmental conditions of summer and winter in the Sahara Desert.

**Results:**

The belowground rhizosheath–root system has higher nutrient and humidity contents, and cooler temperatures than the surrounding sand. The plant responds to the harsh environmental conditions of the summer by increasing the abundance and diversity of extracellular polymeric substances (EPS) compared to the winter. On the contrary, the bacterial community associated with the rhizosheath–root system and its interactome remain stable and, unlike the bulk sand, are unaffected by the seasonal environmental variations. The rhizosheath–root system bacterial communities are consistently dominated by Actinobacteria and *Alphaproteobacteria* and form distinct bacteria communities from those of bulk sand in the two seasons. The microbiome-stabilization mediated by the plant host acts to consistently retain beneficial bacteria with multiple plant growth promoting functions, including those capable to produce EPS, which increase the sand water holding capacity ameliorating the rhizosheath micro-environment.

**Conclusions:**

Our results reveal the capability of plants in desert ecosystems to stabilize their below ground microbial community under seasonal contrasting environmental conditions, minimizing the heterogeneity of the surrounding bulk sand and contributing to the overall holobiont resilience under poly-extreme conditions.

**Supplementary Information:**

The online version contains supplementary material available at 10.1186/s40793-022-00407-3.

## Background

The low levels of moisture and nutrients and general environmental severity make deserts hostile places for plant communities [1, 2]. In order to thrive in deserts, plants have evolved specific morphological and physiological adaptations to optimize water management and nutrient uptake [3–5]. Adaptations of the root system are important to cope with stressful desert conditions. For instance, desert speargrasses collect water condensed from air moisture by the above-ground stems [3, 5] and produce an extensive and prolific root system that rapidly absorb the collected water. Such roots have a unique shape and structure, named rhizosheaths, that increase the retention of absorbed water (“sponge effect”) and reduce the risk of desiccation [6]. The first descriptions of rhizosheaths, defined as “a peculiar sheath, composed of agglutinated particles of sand” critical for tolerance to severe drought, were reported by Volkens [7] and Price [8] in African desert grasses (*Aristida pungens*, *A. obtuse*, and *Lygeum spartum*). Rhizosheath structures have been further reported in several angiosperms, including cereals, herbaceous plants and shrubs [9, 10].

The rhizosheath is a cylindrical, compact, and persistent structure covering the entire root length [11–13]. It is composed of sand particles matted together with root hairs and glued by complex extracellular polymeric substances (EPS) produced by the plant host and the microbial partners. The polysaccharide components of the EPS act as mucigel that, owing to strong absorptive properties, modify the physical architecture of the root–soil zone, enhance the aggregation of sand/soil particles, and concentrate moisture around the rhizosheath retaining up to four-times more water and nutrients than in bulk sand [6, 11, 14–18]. Notably, inoculation of the rhizosheath with EPS-producing bacteria favours soil aggregation around the root and consequently plant growth/tolerance under water deficit and salinity stress [19–21]. In addition, the rhizosheath provides a favourable niche that enriches bacteria and fungi from the surrounding sand [13, 22]. Rhizosheath–root system microbial communities are dominated by desert-adapted Actinobacteria and *Alphaproteobacteria*, and saprophytic Ascomycota fungi, which form stable interactions and carry a broad portfolio of plant growth promoting (PGP) traits and ecological services to the holobiont, including nitrogen fixation and EPS-production [6, 13, 23–27].

The short life cycles of microorganisms, their plastic genomes, and their fast adaptation to fluctuating environments result in a prompt response to abrupt changes in environmental conditions, such as drought [28–30]. This implies that, through their association with the plant, microorganisms can facilitate the host response to stressful conditions [31–33]. An increasing number of studies demonstrated that, despite different soil and/or environmental conditions, plants tend to select a “stress microbiome” [30, 34, 35] that can buffer unfavourable conditions and promote growth [36–39]. However, the extensive surveys on the plant holobiont have been established mainly on a few model plants and on important crops with a vast amount of diversity not yet described for most wild plants [40], such as desert xerophytes. Additionally, limited emphasis has been given to the link between the interactions/associations between plant and microbes under the ecologically-relevant fluctuating environmental conditions occurring in desert ecosystems [41, 42]. We hypothesize that in the rhizosheath–producing xerophytic desert plants the root system metabolism is readapted under contrasting environmental conditions (*e.g.*, seasonal variations) for sustaining and stabilizing the associated bacterial microbiome, and for favouring the overall holobiont resilience in response to perturbations [43, 44]. In this study, we tested this hypothesis by assessing: i) whether a consistent and beneficial bacterial microbiome is retained under the environmental changes occurring over different seasons (summer and winter) in the rhizosheath–root system of *Stipagrostis pungens* (Desf.) De Winter (basionym *Aristida pungens* Desf.), a perennial African speargrass growing on the sandy dunes of the Sahara Desert; ii) whether the microbiome-root holobiont of the rhizosheath system responds to the seasonal environmental changes by modifying its water retention service and the amount of rhizosheath EPS and their chemical nature; iii) whether and how such changes affect the bacterial interactome network of the rhizosheath–root system. Understanding how plants in extreme natural ecosystems manage/control their microbiome (and *vice-versa*) by regulating their physiology/metabolisms (*e.g.*, release of EPS) provide insights into the selective factors that shape the interplay between the holobiont and the surrounding environmental context [45]. Disentangling the plant–microbiome–environment tripartite interaction supports a more efficient exploitation of the plant microbiome resource, as well as contributes to predict the outcomes of global changes on plant–microbe interactions and to develop measures to support wild-desert and desert-farming ecosystems [41, 42].

## Methods

### Study area, sampling and rhizosheath processing

**Fig. 1:**
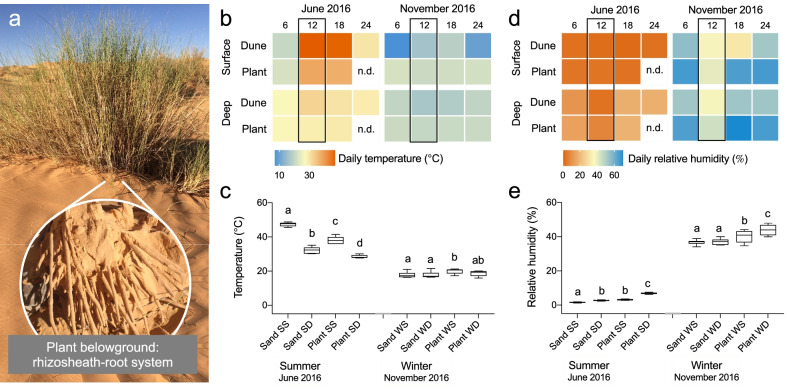
Microclimatic niches in Sahara Desert dune and belowground speargrasses. **a** Representative image of wild speargrass *Stipagrostis pungens* growing in the sandy dunes of the Sahara Desert in Ksar Ghilane, Tunisia; portion of the plant belowground rhizosheath–root system sampled is shown. **b**, **c** Temperature (ºC) and **d**, **e** relative humidity (%) experienced by bulk sand (surface and deep) and belowground plant (*i.e.*, rhizosheath–root system; surface and deep) throughout the day at 6:00, 12:00, 18:00, 24:00 h. Values are expressed as means (*n* = 7). **c** and **e** Box plots showing details of temperature (ºC) and relative humidity (%), respectively, measured at midday (12:00) in barren sand (surface and deep) and belowground plant (surface and deep) in summer and winter; lowercase letters indicate the results of the post-hoc multiple comparison Tukey’s test among sand and belowground plant separately in the two seasons

The sampling campaigns were conducted in the dunes of the Sahara Desert in Ksar Ghilane (Tunisia, 33°00′46.4’’N, 9°37′10.3’’E) during summer and winter seasons in June and November 2016, respectively; we selected these two sampling-times to capture two contrasting environmental conditions of the Sahara Desert ecosystem (annual climatic conditions are provided in Additional file 1: Table S1). In addition, since desert microbial community are ‘time-of-day-dependent’ [46] we sampled between 9:00 and 11:00 am (GMT + 1) in both seasons. African endemic speargrass *Stipagrostis pungens* (Desf.) De Winter (basionym *Aristida pungens* Desf.) was selected as a model plant (Fig. 1a). It is a perennial plant with deep roots and long leaves, drought-resistant, and mainly found in arid and semi-arid regions of North Africa (www.gbif.org/species/4130246) receiving 100–200 mm of rainfall per year [47]. A total of seven speargrass plants of similar size (bunch circumference, 1.9 ± 0.3 m) and active (presence of green leaves) were sampled in each of the two seasons within a dune area encompassing 5 km^2^; plants were sampled at a minimum distance of 100 m apart and did not show visible signs of damage, disease, or human/animal disturbance. The sand surrounding the plants was carefully removed up to 20–40 cm deep in order to uncover the rhizosheath–root system (Fig. 1a) and intact portions were collected (Additional file 1: Fig. S1a) and placed in a sterile tube. Surrounding sand, 5 m away from each plant, was also collected at the same depth (*n* = 7 per season). All samples were collected using sterile tools and plastic containers, stored at –20ºC until processing for DNA extraction and at 4ºC for microbial isolation procedures. In the laboratory, the root-rhizosheath samples were dissected into three different fractions: the rhizosphere, rhizosheath, and root tissue (Additional file 1: Fig. S1b–e). Briefly, the rhizospheric soil weakly attached to the rhizosheath matrix was collected after gently shaking the portions of rhizosheath–root system, then the rhizosheath (compact assemblage of root hairs and sand grains covering the root tissues) was incised and detached from the root tissues; the remaining root tissues were surface sterilized by soaking in 70% ethanol for 3 min, followed by sodium hypochlorite 2.5% for 5 min, 70% ethanol for 30 s, and finally by washing with sterile distilled water five times. The efficacy of the sterilization method was verified by plating pieces of the sterilized root and the water from the last washing step on plates with tryptone soy agar (TSA). The plates were examined for bacterial growth after incubation at 30°C for 3 days.

### Characterization of environmental conditions

Soil and air temperature and relative humidity were measured in situ using weather-proof HOBO U23-001 data loggers during three consecutive days in June (summer) and November (winter) in six locations. Measurements were conducted in the morning (5:30–7:00), at midday (11:30–13:00), in the evening (17:30–19:00), and at midnight (23:30–1:00). The edaphic measurements were recorded at the surface (0/–5 cm) and at depth (–15/–20 cm; hereafter defined as deep) in sandy dunes and under *S. pungens* plants (*i.e.*, belowground under the rhizosheath–root system). Data were analyzed by performing analysis of variance (ANOVA) and Tukey’s multi-comparison tests among the measurements obtained in summer and winter across the different soils (bulk sand and plant belowground) and levels (surface and deep).

### Sand physico-chemical analysis

The chemical and physical properties of the rhizosheath and bulk sand were characterized at Geomar (Germany). Three replicates for each season and type of sample (rhizosheath matrix and bulk sand) were analysed for pH, total carbon (C), organic and inorganic C, total nitrogen (N), organic and inorganic N, available elements/nutrients (nitrite, nitrate, phosphate, and silicate), and texture. The physico-chemical table containing the data from rhizosheath and bulk sand were fourth-root transformed and used to create a resemblance matrix using the Euclidean distance. Significant differences in physico-chemical composition were investigated by permutational analysis of variance (PERMANOVA). PERMANOVA pair-wise tests were also conducted to evaluate the effect of season and fraction. The contribution of the variables to the physico-chemical differences among sites was evaluated by the analysis of similarity percentages (SIMPER). All the analyses were performed in PRIMER v. 6.1 [48].

### Preparation and visualization of rhizosheath–root system samples by scanning electron microscopy (SEM)

For conventional SEM, intact portions of rhizosheath–root systems were rinsed three times with 0.1 M sodium cacodylate buffer (pH 7.2; 15 min each) and fixed in the same buffer containing 1% osmium tetraoxide (OsO_4_) at room temperature in the dark for 1 h. Samples were subsequently rinsed three times with distilled water, dehydrated in an ascending series of ethanol solutions (30%, 50%, 70%, 90%; 15 min each) and finally incubated twice for 15 min in 100% ethanol. Samples were then dried with a critical point dryer (Autosamdri-815B, Tousimis) and mounted on aluminium stubs using adhesive carbon tape and coated with a 5 nm layer of Au/Pb using a K575X sputter coater (Quorum). Samples were observed and imaged with either a Quanta 200 or Quanta 600 FEG SEM, operating with a beam acceleration voltage of 2, 3 or 5 kV. For cryo-SEM, samples were washed in 0.1 M cacodylate buffer and rinsed in distilled water three times before imaging with a Nova Nano SEM (Thermo Fisher Scientific) equipped with a cryo-stage (Quorom technologies). Environmental SEM (ESEM) was also used to determine the elemental composition of sand grains and root material within the rhizosheath–root complex. For this, samples were suspended in distilled water prior to visualisation with a FEI Nova Nano equipped with field emission gun and environmental system. All imaging was performed in the Imaging and Characterization Core Lab at King Abdullah University of Science and Technology (KAUST).

### Fluorescencein situhybridization (FISH) staining of bacteria associated with rhizosheath–root system

Fluorescent in situ hybridization (FISH) was performed on portions of the rhizosheath–root system to observe the distribution of bacteria within the rhizosheath matrix components (root, root hairs and sand grains). The samples were fixed in 4% paraformaldehyde/phosphate-buffered saline (PBS) (3:1 vol:vol) for 12 h at 4°C, washed three times in ice-cold PBS and then stored at –20°C in 1:1 PBS/96% ethanol [49, 50]. After pre-treatment for 10 min with 1 mg mL^−1^ lysozyme and a dehydration series with ethanol at increasing concentrations (50%, 70%, and 96%, 3 min each), an equimolar mixture of the Cy3-labeled probes EUB338, EUB338II and EUB338III was applied for the detection of all bacteria, together with either the Cy5-labelled HGC236 probe (Actinobacteria-specific), the Cy5-labelled ALF968 probe (*Alphaproteobacteria*-specific), or the Cy5-labelled Gam42a probe (*Gammaproteobacteria*-specific) together with the FITC-labelled Bet42a probe (*Betaproteobacteria*-specific) Additional file 1: Table S2. All hybridizations were performed at 40°C for 1.5 h following the protocols described previously [49, 50]; formamide concentrations and properties of FISH probes are reported in Additional file 1: Table S2. After washing steps, stained samples were mounted on glass microscope slides, dried with soft compressed air, and immediately mounted with Citifluor anti-fading medium (AF1; Electron Microscopy Science, Hatfield, USA) before visualization with a Leica TCS SP5 confocal laser-scanning microscope (Leica Microsystems, Mannheim, Germany) equipped with argon and helium/neon lasers. For each field of view, laser intensity, detector settings (gain and offset), and Z-step size (0.15 − 0.5 μm) were optimized to improve resolution and obtain the best signal-to-noise ratio.

### Extraction and quantification of rhizosheath total carbohydrates and extracellular polymeric substances (EPS)

The rhizosheath was detached from root tissues, crushed with a sterile mortar and pestle, and dried. Total carbohydrates (TC), encompassing intracellular and extracellular carbohydrates, were quantified by applying the phenol–sulfuric acid assay [51]. Briefly, 1 ml of 5% phenol was added to 0.03 g dry weight of rhizosheath material, followed by 5 ml of pure H_2_SO_4_ in screw-cap temperate glass vials, stirred for 10 s and left to rest for 10 min. Afterwards, the vials were cooled in cold water for 15 min. Finally, the reaction mix was analysed by determining the absorbance at 488 nm with a Varian Cary 50 UV–VIS spectrophotometer (Varian, Mulgrave, Australia). Calibration was performed using D-glucose at different concentrations as a reference standard and the quantity of carbohydrates expressed as mg of glucose equivalents per gram of rhizosheath dry weight. EPS were extracted from the rhizosheath using a selective approach, adapting the methods reported by Rossi and colleagues [52]. We used distilled water extraction to recover the more soluble and uncondensed EPS fractions of the mucigel (water-extractable EPS, W-EPS). Briefly, 5 ml distilled water was added to 0.1 g rhizosheath DW in screw-cap plastic tubes for 20 min, vigorously shaking to resuspend the rhizosheath material. Then, samples were centrifuged at 5,000⨉ *g* to recover the W-EPS-containing supernatants. Water extraction was repeated three times on each replicate and the W-EPS-containing supernatants pooled for each replicate. To recover the less soluble EPS fractions, more strongly attached to the rhizosheath material, the resulting pellet from the W-EPS extractions was treated with 3 ml 0.1 disodium ethylenediaminetetraacetic acid (Na_2_EDTA) for 20 min. EDTA chelates metal ions bridging the polysaccharidic strands of the EPS, decreasing their aggregation and easing their recovery [53]. The EDTA-extractable EPS (E-EPS) were finally recovered by centrifugation at 5,000⨉ *g*. Na_2_EDTA extraction was repeated three times on each experimental replicate to maximize the extraction efficiency and the E-EDTA-containing supernatants pooled together for each replicate. W-EPS and E-EPS were quantified by applying the phenol–sulfuric acid assay (see above) to 1 ml of W-EPS or E-EPS extracts and then normalizing on the total volume for each extract. EPS fractions were quantified as glucose equivalents, which represented a reference index [52]. For the determination of the monosaccharidic composition of W-EPS and E-EPS, the two fraction were hydrolysed by mixing 1 part extract to 1 part 4 N Trifluoroacetic acid in screw-cap glass vials, for 120 min at 120°C. In the case of E-EPS, prior to hydrolysis, extracts were dialyzed for 24 h against distilled water in 12–14 k MW cut-off nitrocellulose dialysis tubes (Medicell International Ltd., London) to remove Na_2_EDTA in excess which might have interfered with the analytical procedure. The monosaccharidic composition of the polysaccharidic fraction of W-EPS and E-EPS was determined by ion-exchange chromatography (IEC) using a Dionex ICS-500 chromatographer (Dionex, Sunnyvale, CA), equipped with an ion-exchange column (CarboPac PA1) and an ED 50 electrochemical detector with a gold-working electrode. Chromatographic conditions were in accordance with Mugnai and colleagues [54]. The eluents used were Milli-Q-grade water (A), 0.185 M sodium hydroxide (B), and 0.488 M sodium acetate (C). In the first stage of the analysis (from injection time to 7 min), the eluent was constituted by 84% A, 15% B, and 1% C; in the second stage (from 7 to 15 min), the eluent was constituted by 0% A, 50% B, and 50% C; in the final stage (from 15 to 30 min), the eluent was the same as the first stage. The flow was 1.00 mL min^−1^ with running times of 30 min. Response factors of each sugar were determined by injecting known concentrations of pure monosaccharide standards (Sigma-Aldrich).

### Total DNA extraction, sequencing, and bioinformatic processing

Total genomic DNA was extracted from 0.7 ± 0.1 g of crushed sand, rhizosphere and rhizosheath samples using the PowerSoil DNA Kit (Qiagen Inc.) following the manufacturer’s instructions. For root tissues, the genomic DNA was extracted using a DNeasy Plant Maxi Kit (Qiagen Inc.) following the protocols provided by the manufacturer. In total, 56 samples were extracted and stored at –20°C. DNA quantification was performed using a Qubit 3.0 Fluorometer and Qubit dsDNA BR assay kit (Thermo-Fisher Scientific), and quality assessment by electrophoresis on 0.8% agarose gels. We focused our study on bacterial microbiota because preliminary work has shown that the rhizosheath–root system of desert plants hosts a limited amount of fungi [13, 55]. Bacterial libraries were prepared following the two-step dual-indexing approach suggested by Illumina. The V3–V4 region of the 16S rRNA gene was amplified using the primers 341f and 785r [56]. All primers used contained an adapter for the sequencing platform and an 8-nucleotide barcode. A blank control of DNA extraction reagents was also amplified along with a blank PCR to exclude amplification of possible contaminants. Libraries were sequenced on Illumina MiSeq (V3, 300 bp paired-end) at the Biological Core Lab at KAUST. All analyses were performed using the QIIME2, pipeline v2021.2 [57]. First, raw sequences were trimmed and primers removed using cutadapt with default parameters and by removing untrimmed reads [58]. We then used the plugin demux to visualize interactive quality plots and assess read quality, and based on this we truncated the reads at 240 and 200 bp (quality score above 25) for forward and reverse reads, respectively. Using DADA2 with default parameters [59], the reads were denoised and joined to produce amplicon sequence variants (ASVs, average length 417 bp). We further applied the k-mer based alignment-free algorithm ‘KTU’ (K-mer Taxonomic Unit) to re-cluster ASVs into optimal biological taxonomic units [60]. KTUs taxonomy was assigned against the SILVA reference database (v138.1) using the plugin classify-sklearn [61]. The database was trained using the RESCRIPt software [62] and the specific primers sequences that we used. The KTUs unassigned to bacteria (*i.e.*, archaea, unclassified, and plastid) and KTUs present in blank controls were removed from the dataset. All samples presented a suitable sequencing depth (rarefaction curve shown in Additional file 1: Fig. S2) and good’s coverage values (Additional file 1: Table S3).

### Bacterial diversity analyses

Compositional (Bray–Curtis of the log-transformed KTUs table) similarity matrices were calculated and Principal Coordinates Analysis (PCoA) was performed in PRIMER v. 6.1 [48]. Multivariate generalized linear model analysis was performed on the compositional abundance table in R by using *manyglm* function from the package mvabund [63]. The contribution of the explanatory variable to explain the variation of the bacterial community was calculated using the function *best.r.sq* from the same package. The explanatory variables were ‘Compartment’ (4 levels: root tissue, rhizosheath, rhizosphere and bulk sand) and ‘Season’ (2 levels: summer and winter). The components of beta diversity (similarity, replacement and difference in richness) were calculated using the *beta.div.comp* function of the R package adespatial v0.3 [64]. The KTUs table was used to infer the bacterial communities assembly mechanisms occurring in the four compartments across the two seasons by running the phylogenetic bin-based null model (iCAMP) with recommended default settings [65, 66]. Alpha diversity indices (richness and Shannon diversity) were calculated in R using *estimate*_*richness* function in the package phyloseq [67]. Shared and exclusive KTUs (and their relative distribution) across seasons were calculated for each rhizosheath–root system compartment in R using the package VennDiagram [68]; differential abundance of KTUs (twofold-change with *p*-value < 0.001) was also evaluated to determine winter- and summer-enriched KTUs in each compartment by using package DEseq2 in R [69].

### Bacterial co-occurrence network construction

We constructed four individual co-occurrence networks to compare their structure across the environmental niches (*i.e.*, rhizosheath–root system and bulk sand) across seasons (summer and winter). Co-occurrence networks were built using the routine CoNet app in Cytoscape [70] by combining Bray–Curtis (BC) and Kullback-Leiber (KLD) dissimilarity indices, along with the Pearson and Spearman correlation coefficients. Edge-specific permutation and bootstrap score distributions with 1,000 iterations were performed. For each measure and edge, 200 permutations and bootstrap scores were generated and further normalized to detect statistically significant non-random events of co-occurrences. Topological indices of networks were further calculated using the same software; networks were visualized in Gephi [71]. We computed the node degree (the number of edges connecting it to other nodes) and betweenness centrality (relevance of a node in connecting modules) in function of their taxonomic affiliation. We identified the keystone species in each network by ranking the nodes based on the sun of their degree, closeness centrality and betweenness centrality. Co-occurrence networks were reconstructed by applying a correlation-based approach and trophic-interactions were not directly observed.

### Cultivation of bacteria and screeningin vitrofor plant growth promoting (PGP) activity and sand wettability

Bacterial cultivation was conducted starting from 1 g of surface-sterilized root and rhizosheath matrix (*S. pungens* collected in summer) in 9 ml of sterile 0.9% NaCl solution; mixtures were shaken, serially diluted, and plated on solidified oligotrophic media: Reasoner's 2A (R2A) medium (Oxoid) 1 × , R2A 0.1 × , and R2A 1 × with 5% NaCl. The colony-forming units (CFUs) per gram were determined. For each fraction, approximately 50 colonies per medium per fraction were randomly selected, purified, dereplicated by internal transcribed spacer (ITS)-fingerprinting [72] and identified as previously described [38]. The obtained strains were further tested in vitro for their PGP activities, including phosphate solubilization, siderophore release, indole acetic acid (IAA) production, and exopolysaccharide release, along with tolerance to abiotic stresses (temperature: 4°C, 37°C, 42°C, and 50°C; salinity: 5% and 8% of NaCl; and water stress: 20% of Polyethylene glycol—PEG 6000), following the protocols reported by Marasco and colleagues [38].

Four bacterial strains were selected to evaluate their potential role in increasing the water content (WC) of sand: three EPS-positive bacteria (*Enterobacter hormaechei* R12, *Bacillus licheniformis* R56, and *Streptomyces finlayi* R106) were selected as candidates to increase WC of sand, and one EPS-negative bacterial strain (*Pseudomonas putida* R17) as a control. Approximately 30 g (± 0.5) of Sahara Desert sand was distributed in Petri dishes to determine the effect of the selected bacteria. The bacteria were grown in flasks using R2A as medium at 30 °C for 48 h; the cells were harvested by centrifugation at 4000 rpm for 10 min and resuspended in 10 ml of 0.9% NaCl solution to obtain water sand saturation. Finally, bacterial cultures were individually inoculated at a concentration of 10^8^ bacterial cells per g of sand; water without bacterial cells was used as an additional control. The plates were weighed (T_0_) and incubated at room temperature (23°C ± 1°C) for 48 h; plates were weighed every 24 h (T_24_ and T_48_) using a precision balance (NewClassic MF MS204, Mettler-Toledo) and differences between T_0_ were evaluated and defined as relative water content (RWC, %); for each microcosm, the RWC was calculated as (weight T_n_) / (weight T_0_) × 100 (with T_n_ = T_24h_ and T_48h_). To remove the bacterial biomass from the weight of the unevaporated water, we incubated the same quantity of bacterial cells used to inoculate the sand in liquid R2A. These cultures were incubated in the same conditions of sand microcosms and biomass was weighed at 24 h and 48 h using a precision balance (NewClassic MF MS204, Mettler-Toledo) after centrifugation at 4000 rpm for 10 min; the weight of bacterial biomass was subtracted from the weight values obtained from the measurement of sand microcosms in order to obtain the water retained by sand microcosms. The experiment was performed using three replicates per treatment; differences among treatments were tested by using Tukey's multiple comparisons test in GraphPad Prism.

## Results

### Microclimatic niches of Sahara dune and speargrass rhizosheath–root system

The average annual precipitation in the Sahara Desert in Ksar Ghilane, Tunisia ranges between 100 and 200 mm [47]. This ecosystem experiences seasonally different climatic conditions (temperature and relative humidity), along with daily fluctuations (Fig. 1b–e; Additional file 1: Tables S1 and S4). In summer, during the middle of the day, temperatures of dune surface and deep sand reach up to 47.4°C ± 1.2°C and 32.3°C ± 1.9°C, respectively, while in winter lower temperatures are measured (Fig. 1b,c). Relative humidity is also highly variable across seasons, ranging between 1.5% and 17% in summer and increasing up to 50% in winter (Fig. 1d). Soil temperatures in the deep and surface parts under the plant are almost 10°C and 4°C less, respectively, than those measured in the dune sand without plants (Fig. 1c). In winter, due to the milder conditions of the desert climate, differences between the temperature of sand and plant-belowground were detected only for the superficial soil (Fig. 1c). Similarly, the belowground rhizosheath–root system increased the soil moisture, consistently maintaining higher values of relative humidity than in the dune sand (Fig. 1d,e), with values almost 2.1-fold and 2.5-fold higher than those of dune sand in surface and deep layers, respectively, during the hot and dry summer (Fig. 1e).

Along with stabilization of temperature and humidity, the rhizosheath–root system showed significantly different physico-chemical conditions compared to the bulk sand in both summer and winter (Additional file 1: Tables S5 and S6). Nutrients (organic C and N) were significantly enriched compared to the oligotrophic sand (Additional file 1: Table S5); organic C content in the rhizosheath was higher under dry conditions (summer) with an eightfold increment compared to bulk sand and a 1.5-fold increment compared to winter rhizosheath (Additional file 1: Table S5).

### Microbial colonization and morphology of rhizosheath matrix of *S. pungens*

**Fig. 2:**
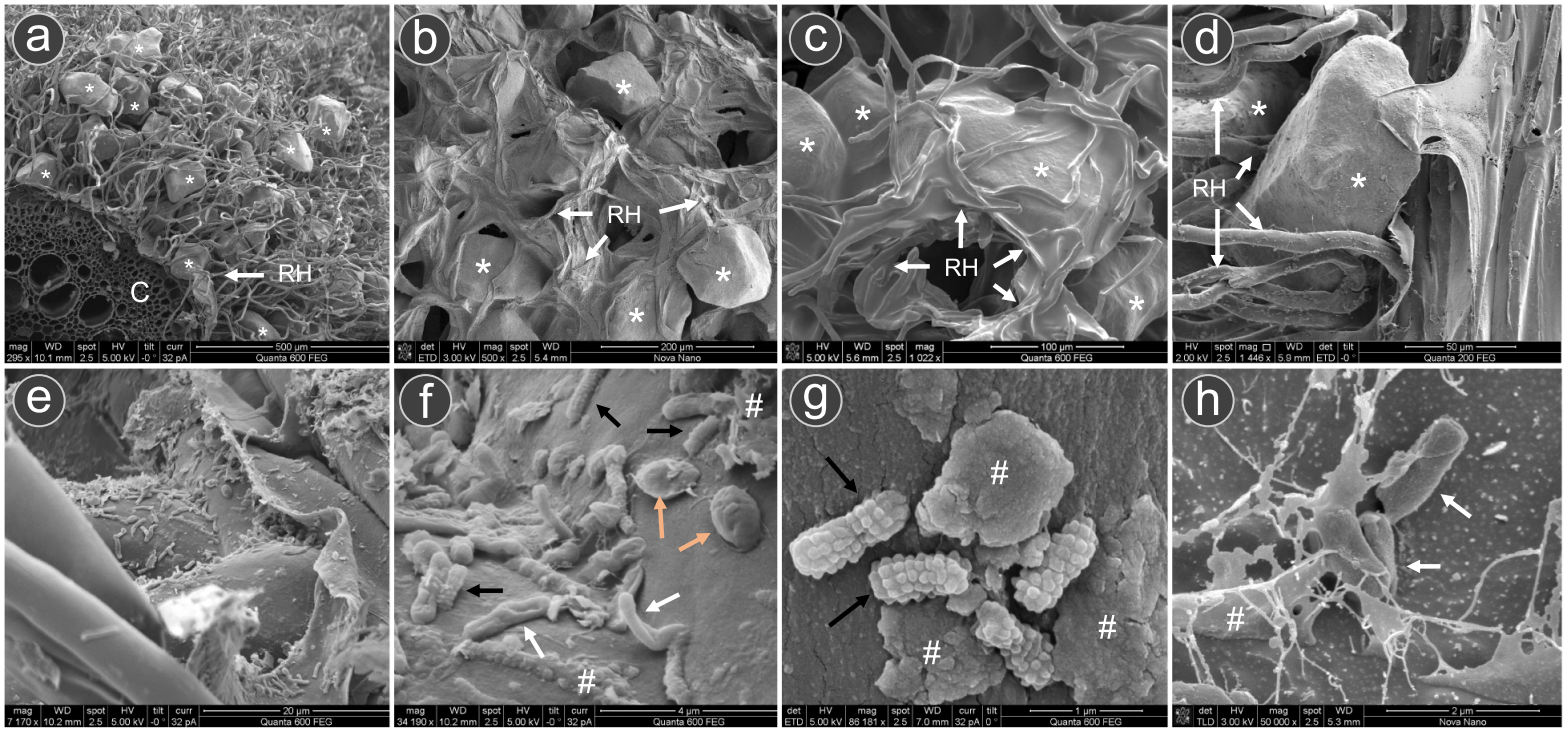
Visualization of *Stipagrostis pungens* rhizosheath with scanning electron microscopy (SEM). **a** Scanning electron microscope (SEM) image of a rhizosheath–root transverse section showing a dense matrix of root hairs (RH) and sand grains (asterisk, *) covering the central root cortex (**c**); separation between the epidermis and cortex (cortical lysis) is visible in Supplementary Fig. S2a. **b**–**d** Magnification of the rhizosheath matrix showing sand grains (asterisk, *) with intertwined root hairs (RH) and interstices, coated with mucilages (cryo-SEM panels **b**, **c**, and Supplementary Fig. S4a; SEM panel **d**, and Supplementary Fig. S4b) and flaky material on both sand grain and root hair surfaces (SEM in Supplementary Fig. S4c–i). **e**–**h** SEM images showing bacterial cells associated with the surface of roots, root hairs and sand grains (refer also to Supplementary Fig. S4). Note the different scales on the SEM micrographs; arrows with different colours indicate some of the different bacterial cells observed: black, lobed/warty surface; white, rods; orange, coccobacillus; #: EPS materials

The exterior portion of the rhizosheath–root system was a compact cylinder composed of sand grains (Additional file 1: Result S1) and root hairs surrounding the epidermal surface along the entire length of the root (Fig. 1a and 2a; Additional file 1: Figs. S1a,b and S3a). The sand grains were physically entrapped in a dense net of root hairs (Fig. 2a–c) and were covered/stabilized by an EPS matrix with the appearance of a binding/coating mucilage and flaky material (Fig. 2b–d; Additional file 1: Figs. S3a,b and S4). High prokaryotic cells numbers attached to all the components of the rhizosheath matrix were detected (Fig. 2e–h; Additional file 1: Fig. S3c–n). Most of the bacterial cells observed were rod-shaped and spherical-shaped, forming cell clusters on the surface of roots, root hairs and sand grains (Fig. 2e–h; Additional file 1: Fig. S3c–e). Microbial cells mainly lay on the surfaces of rhizosheath components, while some cells were perpendicularly oriented; some of the cells also presented visible wires or peduncles for anchoring (Fig. 2f; Additional file 1: Fig. S3i). Along with rods and cocci, we detected aerial hyphae possibly produced by members of Actinobacteria (Additional file 1: Fig. S3f,m,n) and several bacterial cells with unusual morphology, including bacteria with stalk-like forms and lobed/warty surface, polysporous actinomycetes and coccobacilli-like cells (Fig. 2f,g; Additional file 1: Fig. S3g-m). The presence of a complex bacterial community was confirmed by FISH-CLSM microscopy (Additional file 1: Fig. S5). The Actinobacteria-specific probe revealed their majority and ubiquitous presence on root hairs and sand grain surfaces both as single cells and clusters of cells with frequent cell–cell interactions with other bacteria (Additional file 1: Fig. S5). Notably, we were not able to detect mycorrhizal fungal hyphae or other microeukaryotic-sized cells (*e.g.*, yeasts) within the rhizosheath matrix or its surface.

### Quantification and characterization of total carbohydrates and EPS in the rhizosheath matrix of *S. pungens* across seasons

The rhizosheaths collected in two contrasting seasons, namely summer and winter, were characterized by a similar (*p* > 0.05) content of total carbohydrates (TC; Table 1). Among TC, the EPS—encompassing W-EPS and E-EPS—showed significant seasonal quantitative variation (t_1,4_ = 4.316, *p* = 0.012), with a twofold increment in summer compared to winter (respectively 0.15 ± 0.01 *vs.* 0.073 ± 0.01 mg/g DW; Table 1). This change was driven by the W-EPS fraction (t_1,4_ = 5.939, *p* = 0.004) and not by the E-EPS fraction (t_1,4_ = 1.552, *p* = 0.19). W-EPS represented 9.14% and 4.35% of the TC content during summer and winter, respectively, while E-EPS represented 2.2% and 1.03% of the TC content, respectively (Table 1). IEC analysis revealed that the rhizosheath EPS contained up to 11 different monosaccharides (Table 2). Glucose, xylose, galactose, and arabinose were the most represented monosaccharides, roughly constituting 93–95 mol% of the total monosaccharidic composition. However, we detected significant differences in their distribution according to the EPS fraction (W-EPS or E-EPS) and sampling season (Table 2). Within the W-EPS fraction we had a higher amount of glucose in winter compared to summer (54.5 and 37.4 mol%, respectively), while the opposite trend was observed for arabinose (5.23 and 9.54 mol% in winter and summer, respectively) and xylose (16.77 and 28.04 mol%, respectively). Fucose, rhamnose and ribose were detected only in the W-EPS fraction extracted from the summer rhizosheath, with rhamnose detected exclusively in this EPS fraction. In the case of the E-EPS fraction, we observed a monosaccharidic composition similar to that of the W-EPS fraction in the two seasons (Table 2). Notably, ribose—present in both the W-EPS and the E-EPS fractions—was detected only in the rhizosheath collected in summer (Table 2).

**Table 1:** Total carbohydrate (TC), water-extractable EPS (W-EPS) and EDTA-extractable EPS (E-EPS) contents in mg glucose equivalents g^−1^ DW of rhizosheath of speargrasses collected in summer and winter

Season	TC	W-EPS	E-EPS	% (W-EPS/TC)	% (E-EPS/TC)
Summer	1.36 ± 0.32 (a)	0.12 ± 0.01 (a)	0.03 ± 0.02 (a)	9.14	2.22
Winter	1.08 ± 0.09 (a)	0.06 ± 0.02 (b)	0.01 ± 0.01 (a)	4.35	1.03

Percentages of W-EPS and E-EPS content over TC content are also reported. All values are expressed as mean ± SD calculated on three experimental replicates. Significant differences between seasons (summer *vs.* winter) are indicated by different lower-case letters (*t*-test, *p* < 0.05)

**Table 2:** Monosaccharide composition of W-EPS and E-EPS extracted from rhizosheaths sampled in summer and winter

Sugar	Abbr	W-EPS (moles %)		E-EPS (moles %)	
		Summer	Winter	Summer	Winter
*Arabinose*	Ara	9.54	5.23	11.91	9.55
*Fructose*	Fru	n.d	n.d	0.84	2.96
*Fucose*	Fuc	0.67	n.d	0.94	0.67
*Galactose*	Gal	10.14	10.98	15.19	10.14
*Galactosamine*	GalN	n.d	n.d	n.d	0.60
*Glucose*	Glc	44.22	64.69	30.50	44.24
*Glucosamine*	GlcN	3.78	2.34	2.67	3.78
*Rhamnose*	Rha	0.66	n.d	n.d	n.d
*Ribose*	Rib	2.96	n.d	1.44	n.d
*Xylose*	Xyl	28.04	16.77	36.50	28.06

Values are expressed as moles of the single monosaccharide divided by the total number of moles of monosaccharides in W-EPS and E-EPS × 100); data shown are mean values from at least three replicates; standard deviations never exceeded 5%; n.d., not detected

### Niche partitioning of bacterial community across rhizosheath–root system compartments

**Fig. 3:**
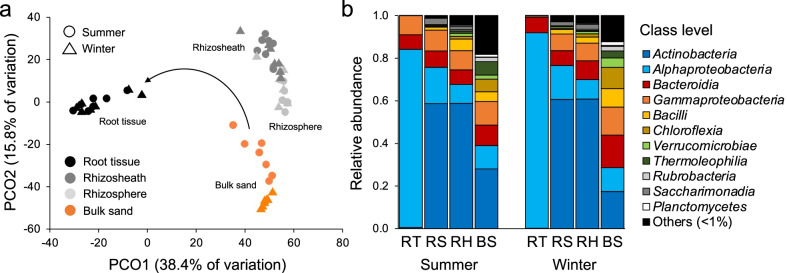
Bacterial community diversity and composition across rhizosheath–root system compartments and seasons. **a** Ordination based on principle coordinates analysis (PCoA) using Bray–Curtis dissimilarity for bacterial microbiota associated with root (RT), rhizosheath matrix (RS), rhizosphere (RH), and bulk sand (BS) compartments in summer and winter seasons (*n* = 56). Colours denote compartments, and shapes denote seasons (summer and winter). **b** Bacterial microbiota composition across compartments in summer and winter; relative abundance of the main phyla/classes (relative abundance > 1%) is reported as mean of replicates (*n* = 7) for each category (compartment per season). Taxonomic groups present with relative abundance < 1% are summed and reported as “Others”

Bacterial diversity of the rhizosheath–root system (root tissues, rhizosheath matrix, and rhizosphere; *n* = 42) and bulk sand samples (*n* = 14) were studied using 16S rRNA gene sequencing. A total of 3,339,366 high-quality sequences classified in 2,028 KTUs were obtained. The sequencing effort was sufficient to capture the most abundant and rare taxa (Additional file 1: Fig. S6). The bacterial microbiota was firstly influenced by the plant compartments (generalized multivariate linear model test: GLM_3,52_ = 43,963, *p* = 0.001; Table 3; pairwise comparison in Additional file 1: Table S7), following a clear niche-partition driven by plant selection. Principal Coordinates Analysis (PCoA) based on BC dissimilarity matrix highlighted this significant spatial niche separation (Fig. 3a; KTU distribution in Additional file 1: Fig. S7) with the primary axis (38.4% of total variance) distinguishing edaphic communities (rhizosheath matrix, rhizosphere, and bulk sand) from the root endophytic communities, and the secondary axis (15.8%) separating edaphic communities associated with the plant (rhizosheath matrix and rhizosphere) from those of bulk sand. The bacterial communities of the four compartments displayed heterogenous dispersion (PERMDISP: F_3,52_ = 6.11, *p* = 0.007): while both rhizospheric and rhizosheathic bacterial communities were more similar to each other (low dispersion), those in bulk sands had higher dispersion values (Additional file 1: Table S8); notably, the highest values of dispersion were observed in the root endophytic communities, possibly due to selection processes linked to other plant-related factors not assessed in this work (*e.g*., plant age). By applying the null model based on iCAMP analysis [65], we found that drift (range, 7.9%–66.7%), dispersal limitation (5.6%–33.4%) and selection (9.1%–84.8%) were the main processes that drive the assembly of the bacterial communities associated with the rhizosheath–root system and the bulk sand, along with homogenizing dispersal that was mainly observed in bulk sand (up to 13.6%; Additional file 1: Table S9). The relative contribute of these deterministic and stochastic processes varied along the four compartments (Cohen’ D test, *p*-value < 0.05 for 85% of the comparisons), suggesting that different mechanisms of assembly are taking place in these niches. Alpha-diversity indices (within-sample diversity) indicated a gradual decrease of bacterial diversity from bulk sand to root tissues (richness: F_3,52_ = 29.9, *p* < 0.0001; Shannon diversity: F_3,52_ = 254, *p* < 0.0001; Additional file 1: Fig. S8). At phylum/class rank, edaphic communities were dominated by Actinobacteria (47.4% ± 19.4%), Proteobacteria (12.2% ± 3.5% *Alphaproteobacteria* and 9.8% ± 2% *Gammaproteobacteria*), Bacteroidia (9.2% ± 3.2%), and several other minor groups (defined as “others” in Fig. 3b), while root tissues were colonized by Proteobacteria (87.6% ± 5.8% *Alphaproteobacteria* and 4.8% ± 5.9% *Gammaproteobacteria*) and Bacteroidia (7.1% ± 0.3%; Fig. 3b; Additional file 2: Data S1).

**Table 3:** Quantification of microbial community variability explained by each factor (compartment, season, and compartment across season); results of multivariate tests and R^2^ are reported

Factor	Multivariate test	*R*^2^ (%)
Compartment	Dev_3,52_ = 43,963, *p* = 0.001*	27.3
Season	Dev_1,54_ = 4187, *p* = 0.024*	3.86
Compartment across seasons		
Root tissue	Dev_1,12_ = 127, *p* = 0.131	7.96
Rhizosheath	Dev_1,12_ = 2014, *p* = 0.059	10.2
Rhizosphere	Dev_1,12_ = 2166, *p* = 0.065	9.86
Bulk sand	Dev_1,12_ = 9971, *p* = 0.001*	27.8

Star (*) indicates statistical significance, *p*-value < 0.05

### Seasonal effects on the rhizosheath–root system bacterial microbiome

We did not detect significant changes in the composition of the bacterial microbiome associated with the root tissue, rhizosheath matrix, and rhizosphere across the two seasons (Table 3). On the contrary, bulk sand (not subjected to the plant root selective-pressure) hosted significantly different bacterial microbiomes, with the factor season explaining 27.8% of the observed variation (Table 3; Fig. 3b; results of univariate test in Additional file 1: Table S10); the unexplained variation could be ascribed to other unmeasured environmental factors. Based on the analyses of betadiversity components, the diversity among summer and winter bulk sands was mainly determined by a richness difference (44%; Additional file 1: Fig. S9). Notably, the assembly of the bulk sand community during the winter season had a higher relative contribution of homogeneous selection and dispersal limitation, and lower relative importance of drift when compared with those in summer (Cohen’s D test, *p*-values < 0.05; Additional file 1: Table S9). This result suggests that within the bacterial communities of bulk sand certain populations are under strong selection (*e.g.*, bins belonging to *Burkholderiales*, *Kallotenuales*, *Cytophagales* and *Bacillales*) whereas others are under strong drift (*e.g.*, bins affiliated to *Actinobacteria* and *Propionibacteriales* orders within the Actinobacteria phylum), and that their ratio changes across the two seasons defining different assembly and communities. Differences in the bacterial community of bulk sand across the seasons were also detected in terms of alpha-diversity; the overall KTU richness decreased with increasingly stressful conditions (Additional file 1: Fig. S10a); a significant negative correlation between richness and temperature was detected (F_1,11_ = 27.16, *p* = 0.0003, R^2^ = 0.71). No significant differences in Shannon diversity were detected (Additional file 1: Fig. S10b), possibly because despite a change in the community members the type of distribution (*e.g.*, dominance relations) remained constant.

### Characterization of shared and unique bacterial components across seasons

**Fig. 4:**
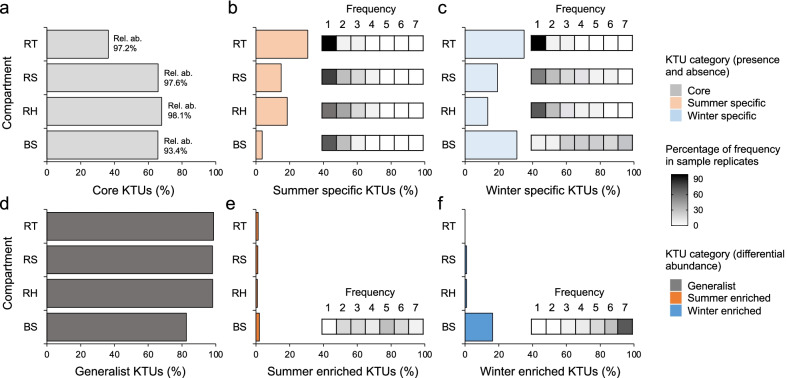
Distribution of bacterial KTUs in bulk sand and rhizosheath–root system compartments across two contrasting seasons. **a**–**c** Percentage of bacterial KTUs present in **a** both seasons (core), and in **b** summer or **c** winter only (summer-specific and winter-specific, respectively) in each compartment (RT, root tissue; RS, rhizosheath; RH, rhizosphere; BS, bulk sand). Percentage of frequency (number of samples that contain that KTU, min = 1, max = 7) is reported for each KTUs category (core, and summer and winter specific). **d**–**f** Percentage of KTUs showing **d** similar relative abundance across seasons (generalist) and that have a differential abundance in (**e**) summer or (**f**) winter (season specific; twofold change average relative abundance and *p*-value < 0.01) are reported for the four compartments; details regarding the frequency of these KTUs are reported only for bulk sand—the only compartment showing an elevated percentage of KTUs with differential abundance across seasons

We consistently found a seasonal core bacterial microbiome in all compartments, accounting for 68% and 66% of the KTUs in the rhizosheath and rhizosphere, respectively, 65.7% in bulk sand and 30% in root tissues (Fig. 4; Additional file 1: Fig. S11). The seasonal core bacterial microbiomes were composed of the most abundant KTUs in all compartments, with percentage of relative abundance ranging from 98.1% in the rhizosphere to 93.4% in bulk sand. Compartments also showed season-specific KTUs; while they were limited, in terms of number and abundance, in the rhizosheath matrix and rhizosphere, these KTUs constituted an important portion in bulk sand and root tissues (Fig. 4b,c). In bulk sand these KTUs were particularly abundant in winter and accounted for 30.7% of the KTUs and 6.4% of the relative abundance. In the case of root tissues, season-specific KTUs were high in terms of number (total, 70% of the KTUs) but low in terms of relative abundance (2.9%). The majority of the season-specific KTUs (58.4% of winter-specific) in bulk sand were detected in at least 4 of the 7 replicates, while in the root tissues these KTUs were randomly distributed across single replicates (95% percentage of KTUs were detected in only one sample; Fig. 4b,c).

We further quantified the number of differential abundant KTUs in each of the four compartments across the two seasons (*p* < 0.01 and twofold changes in relative abundance; Fig. 4d–f). Only a small number of KTUs was differentially accumulated in summer and winter when root tissue, rhizosheath matrix, and rhizosphere were considered (Fig. 4e, f). On the contrary, in bulk sand the bacterial components significantly changed in their relative abundance over seasons, 21 and 309 (of 1,895) in summer and winter, respectively (Fig. 4e ,f; Additional file 1: Fig. S12). While the winter-enriched KTUs comprised taxa belonging to the *Bacteroidia, Verrucomicrobia*, *Gammaproteobacteria, Alphaproteobacteria, Chloroflexia*, *Bacilli*, *Bdellovibrionia*, *Thermoleophilia*, *Polyangia* and *Saccharimonadia* (ordered by decreasing abundance), KTUs from the summer-enriched group were mostly from *Actinobacteria*. It is important to note that although changes in relative abundance can be interpreted as changes of specific KTUs, they could be the result of decreases/increases in other community members rather than, or in addition to, changes in their absolute abundance.

### Bacterial interactions in the rhizosheath matrix and bulk sand across seasons

**Fig. 5:**
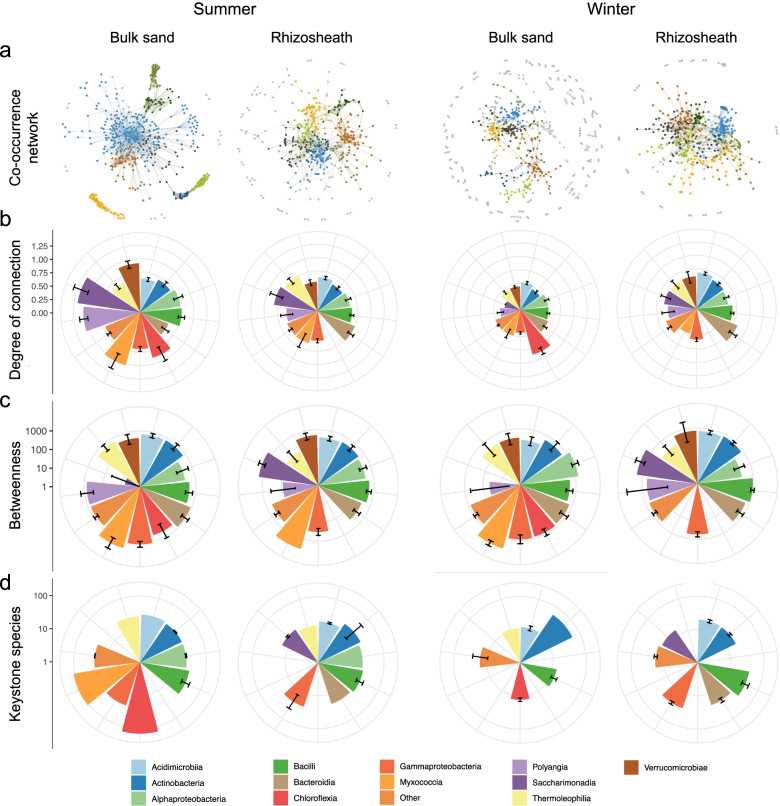
Co-occurrence correlation network of the bacterial communities associated with rhizosheath–root system and bulk sand in contrasting seasons. **a** Visualization of co-occurrence bacterial networks of rhizosheath–root system and bulk sand in summer and winter. Large modules with ≥ 3% of nodes are shown in different colours, and smaller modules are shown in grey; colours are assigned randomly. Network topological attributes are listed in Table 4. **b** Degree of connection of nodes within each network; results are visualized at the bacterial class level and expressed as Log-transformed average. **c** Betweenness centrality of nodes calculated for each co-occurrence network; results are visualized at the bacterial class level and expressed as Log-transformed average. **d** Visualization of the first 20 keystone species identified based on the sum of degree, closeness centrality and betweenness centrality for each node in the four networks; results are visualized at the bacterial class level and expressed as Log-transformed average

To identify potential interactions among bacterial microbiome members associated with the rhizosheath–root system and bulk sand, we constructed co-occurrence networks for each season (Fig. 5). Co-occurrence networks were composed of nodes constituting between 61% and 75% of the total KTUs (Table 4). In the rhizosheath–root system networks, nodes were mainly connected by positive correlations, while those in bulk sand had higher negative correlations between nodes, reaching 42% and 51% in summer and winter, respectively (Table 4). The bacterial networks in the rhizosheath–root system and bulk sand showed different structures (Table 4), as well as significantly different betweenness centrality (a measure of the influence exerted by an KTU over the network; ANOVA: F_1,1918_ = 7.7558, *p* = 0.0054) and degree centrality (a measure of the level at which an KTU co-occurs; F_1,1918_ = 7.015, *p* = 0.0081). Notably, bacterial networks in the rhizosheath–root system had a similar structure across seasons, which was not the case in bulk sand (Table 4; Additional file 1: Table S11). For instance, in summer the bulk sand community had a significantly higher connectivity among nodes (Tukey HSD pairwise comparison, *p* < 0.0001; Fig. 5b) and of average node connectivity, and, at the same time, a smaller diameter and path length than those in winter (Table 4). In summer the bulk showed also a higher heterogeneity and centralization, along with a differential betweenness centrality (*p* < 0.0029; Fig. 5c; Additional file 1: Table S11). Analysis of the taxonomic composition of rhizosheath–root system networks showed that nodes belonging to *Chloroflexia*, *Rubrobacteria* (refer to others), *Bacteroidia*, *Alphaproteobacteria* and *Actinobacteria* were the main interactors in both seasons (Fig. 5b,c; Additional file 1: Fig. S13), with members of *Actinobacteria*, *Bacteroidia* and *Chloroflexia* consistently identified as keystone species (Fig. 5d). In the case of bulk sand, members of *Myxococcia*, *Clostridia*, *Saccharimonadia*, *Polyangia*, *Verrucomicrobia* and others (including, *Fusobacteriia* and *Elusimicrobia*) had the higher degrees of connection during summer, while during winter the connections were mainly built by *Clostridia* followed by the remaining taxa that showed an equal distribution of degrees (Fig. 5b; Additional file 1: Fig. S13). Similarly, keystone species of the bulk sands networks were taxonomically different among the two seasons (*p* = 0.0078; Fig. 5d; Additional file 1: Table S11).

**Table 4:** Topological properties of the co-occurrence networks of bacterial communities associated with rhizosheath root system (RS) and bulk sand (BS) in summer and winter seasons

Topology	Summer RS	Winter RS	Summer BS	Winter BS
Nodes (network size)	429	442	468	583
% of initial KTUs (> 0.01%)	73	75	61	63
Connectivity (total number of interactions)	958	1031	1654	810
N. Positive (% of total)	627 (65.4%)	730 (70.8%)	961 (58%)	397 (49%)
N. Negative (% of total)	331 (34.6%)	301 (29.2%)	693 (42%)	413 (51%)
Av. connectivity (av. interaction per node)*	4.47 a	4.66 a	7.1 b	2.78 c
Av. positive*	2.92 a	3.31 a	4.11 b	1.36 c
Av. negative*	1.54 a	1.36 a	2.96 b	1.41 a
Av. neighbours	4.807	4.936	6.671	3.552
Diameter	13	14	14	23
Path length	4.29	4.58	4.41	7.05
Cluster coefficient	0.280	0.262	0.255	0.271
Density	0.012	0.012	0.016	0.009
Heterogeneity	1.149	1.087	1.638	1.283
Centralization	0.127	0.106	0.322	0.136
Connected components	18	15	9	78
Modularity^#^	0.701	0.676	0.756	0.838
Modules^#^	29	24	16	91

^*^Letters indicate the results of Tukey’s multiple comparisons test, significance *p* < 0.05

^#^Calculated in Gephi using the default parameters

### Cultivable bacteria and their functional role in the rhizosheath–root system

Bacterial isolates (Additional file 1: Result S2) were tested in vitro for plant growth promoting (PGP) traits and tolerance to abiotic stresses typical of desert environments (drought, salinity, fluctuating temperature; Additional file 1: Table S12). 63% of total bacteria were involved in biopromotion activity (indole-acetic acid [IAA] production), while among rhizosheathic bacteria 20% were possible biofertilizers (showing at least one activity among siderophore and phosphate solubilization), and 66% possible drought-bioprotectors due to their capacity to produce EPS. Bacteria also showed a high tolerance toward abiotic stresses with 100% and 70% of the rhizosheathic and endophytic strains, respectively, actively growing under water limitation (20% of PEG), 60% and 1.5% able to grow in presence of salt (8% of NaCl), 13.5% and 1.5% tolerating high temperature (50°C; Additional file 1: Table S12). We further evaluated whether EPS-producing bacteria from the rhizosheath matrix could retain water in sandy substrates. Results of incubation (48 h) after initial irrigation (100% water saturation) showed that two out of the three EPS-producing bacteria, R12^EPS+^ (*Enterobacter hormaechei*) and R56^EPS+^ (*Bacillus licheniformis*), were able to increase water retention in the sand of 3.3% and 3%, respectively, compared to the control (NC) not treated with bacterial cells (*p* < 0.0001) and the EPS^–^ strain R17 (*Pseudomonas putida*; *p* = 0.0001 and *p* = 0.0003, respectively; Additional file 1: Fig. S14a). They showed consistently higher water content values throughout the incubation time (Additional file 1: Fig. S14b). The presence of R106^EPS+^ (*Streptomyces finlayi*) with water-retention values similar to the NC (*p* > 0.05) indicated that not all the EPS producers have the same effect on water retention in sand.

## Discussion

In desert and other dryland soils, plants and their associated microbiomes have to cope with multiple stresses, such as high irradiation and water deficiency, and environmental variations, such as daily and seasonal fluctuations of temperature and humidity [1, 73]. These environmental conditions restrict plants and microbial activity and act as selective forces regulating plant–soil–microbe interactions [74]. In particular, the environmental conditions of deserts, and those of the plant host, play an active role in selecting microbial communities and populations that can mediate acclimation and adaptive responses of plants to stresses [75–77]; for example, soil microbial communities historically exposed to drought sustain plant resistance to water stress [31, 33]. For this reason, the preservation of an active, cooperative, and consistent interaction between plants (provision of nutrients) and microbes (alleviation of plant stress) is an advantage for both components [40, 78]. Therefore, understanding the interactions between bacteria and desert-adapted plants, and the recruitment strategies mediated by the latter, is important in the prediction of the impact of desertification on vegetation, as well as management strategies for soil restoration [9, 12, 16, 17]. This is of even greater importance considering the fact that under ongoing climate change, soil desertification and dryland areas will increase by 11%–23% by the end of this century with profound and lasting impacts on the associated microbiomes [1, 77, 79].

### EPS content and type in the rhizosheath matrix change over seasons

A poorly studied adaptive trait of plants to cope with drought is the formation of a rhizosheath matrix, mainly developed by *Poaceae* species (wild and cultivated) under dry soil conditions [9, 11, 13, 80]. The mechanisms involved in the formation and stabilization of this matrix are not fully elucidated. Several studies proposed that it is the result of a combination of factors, including the morphological and physiological characteristics of the root system, the associated microbiota, and the environmental conditions [25, 80–83]. For example, during extended drought periods, some grass species increase the thickness of their rhizosheaths [84, 85], mainly stimulating the production of mucilage (*e.g.*, xyloglucan) to enhance water infiltration and aeration [86]. Modulation of EPS production under stress was also observed in the *S. pungens* rhizosheath matrix studied here. The EPS polysaccharidic composition was dominated by glucose, galactose, arabinose, and xylose. The presence of the latter two monosaccharides strongly suggests a plant origin of part of the EPS mucigel [87], while the scarcity of non-neutral sugars and the absence of glucuronic and galacturonic acids suggest the limited contribution of cyanobacteria and microalgae to the EPS matrix of the rhizosheath [88, 89]. Overall, the EPS content showed a 50% increment from winter to summer, indicating a possible physiological adaptation of the plant-microbiota holobiont to the overall increase of temperature and decrease of relative humidity in the hot/dry season. The release of a higher amount of EPS enhances moisture maintenance against evaporation, and contributes to water capture from non-rainfall sources, such as dew, fog, and plant guttation [5, 90]. In addition, based on microcosm studies under drought stress, the increment of the sole soluble EPS fraction, as we also observed, was associated with an increase in surface water repellence, which reduced water movement and its dispersion in the sand [91]. The dominance of the most soluble fraction of EPS (W-EPS) also represents a readily available and easily assimilable carbon reservoir for the edaphic microbiota [92], including heterotrophic members [54, 93] when favourable conditions for microbial growth in the soil are available. Along with the overall increase in the amount of EPS, the hot/dry season also stimulated the production of a more complex mix of less-soluble EPS (E-EPS) that can be more condensed and more tightly bound to bacterial cells, sand, and plant tissues [52, 94]. The presence of the deoxy sugars fucose, rhamnose, and pentose ribose confers an amphiphilic quality to the rhizosheath; in other words, both ionic bonds and non-polar interactions can be established, favouring cell adhesion and cell aggregation by removing the water film between the cell surface and the substrate [95]. This more condensed EPS fraction, owing to lower solubility and diffusion, is better preserved from microbial activity [93] and may have an important binding/structural role in the rhizosheath matrix, as well as in regulating water uptake and loss [92, 96]. It may provide a more protected/stable niche for microorganisms under the harsh environmental conditions of the summer desert.

### Bacterial communities in the rhizosheath matrix are stable over seasons

In the bulk sand the bacterial communities differ between seasons due to a differential combination of deterministic and stochastic factors (Cohen’s D test, *p*-values < 0.05, refer to Additional file 1: Table S9) that interact to diversify the bacterial assembly pattern and the taxa turnover (*e.g.*, Actinobacteria dominates in summer, while *Chloroflexia*, *Bacteroidia*, and *Gammaproteobacteria* in winter). Conversely, the rhizosheath–root system bacterial microbiome was characterized by consistent bacterial communities in both seasons. Although changing over time as a result of modifications in the plant’s physiology (summer *vs.* winter; Table 1 and 2), the rhizosheath–root niche select for a consistent and reduced bacterial diversity (lower richness and Shannon diversity compared to bulk sand; Additional file 1: Fig. S8), following the same combination of ecological drivers (Cohen’s D test, *p*-values > 0.05 for each process; Additional file 1: Table S9) that result in the homogenization of the bacterial communities’ compositional profile (Fig. 3 and Table 3). Based on these results, we propose that the holobiont adapts to the changing environmental conditions of the different seasons by regulating the EPS metabolism rather than restructuring the microbial community diversity. This phenomenon is widely observed in the bacterial and plant world: abiotic environmental factors can modulate the productivity and composition of microbial EPS and root exudates [54, 97], driving the adaptation of (micro)organisms and plant-microbiome feedbacks [45]. Along with the stabilization mediated by EPS/root exudates, the detritus associated with the preceding/decaying roots also may play an important role in selecting the microbiome of the root systems [98]. For example, in the presence of decaying roots wheat and chickpea rhizosphere microbiomes were homogenous (65%–87% similarity), while disruption by tillage increased microbiome heterogeneity (3%–24% similarity). These results suggest that in perennial plant species, like *S. pungens*, the new roots can be strongly influenced by the surrounding old/decaying roots that act as a “selected microbial reservoir”; consequently, this bacterial pool previously selected by the plant can be “recycled” over time, driving homogenization of the rhizosheath bacterial microbiome and soil micro-niches across seasons [98, 99]. In the bulk sand, where the plant/rhizosheath legacy is absent, the effect of seasonality and heterogeneity on the bacterial microbiome are evident, and mainly driven by the environmental conditions, such as water availability [100, 101]. However, other studies conducted on non-perennial and non-rhizosheathic desert plants associated with the Atacama Desert bloom events (*e.g*., *Cistanthe longiscapa*) detected a seasonality also in the rhizosphere-associated microbiota [76], suggesting that microbial stabilization is a peculiar feature of rhizosheathic desert plants.

### Implications of an environmentally-independent rhizosheath microbiome

The presence of a stable and consistent bacterial community associated with the rhizosheath–root system suggests that the plant creates the conditions to select an environmentally-independent bacterial microbiome [102], with two possible implications. On the one hand, an environmentally-independent bacterial community can reduce the sensitivity to environmental changes and favour the holobiont resistance due to a consistent taxonomic and functional diversity of the microbiome [30, 31, 33] that can be maintained as long as the plant can cope with the predicted stress. Indeed, if we consider that any loss in microbial diversity, such as that observed in bulk sand during summer, will likely reduce multiple ecosystem functions and services [103], we can interpret the rhizosheath–root system stabilization of the microbiome, in terms of richness and composition, as a consistent provision of beneficial services over time (*i.e.*, biofertilization and biopromotion detected among the bacteria cultivated here) that can actively support the resistance capacity of the holobiont. On the other hand, an environmentally-independent bacterial community tends to establish a stable bacterial interactome, counteracting the disaggregating effects that have been observed during environmental changes, such as aridity intensification and warming [77, 104], and maintaining the functional plant–microbe mutualism/antagonism ratio established by the holobiont regardless the climate context [105]. Since the networking among bacterial community members has strong linkages with ecosystem functioning [106], the preservation of its structure is also important for the conservation of functionality and services within the bacterial microbiome of the rhizosheath–root system. In our case, rhizosheath networking was dominated by positive correlations with cooperative behaviours, such as cross-feeding, commensalism, syntrophic and mutualistic interactions [77], that contribute to maintain the stability of the system over time and its resistance to environmental fluctuations. However, in a scenario in which the plant microbiome cannot be resistant to adverse changes and stresses, such as extended drought and unexpected heat waves, the high level of cooperative behaviours, the physiological flexibility and the functional redundancy observed make it plausible that such a microbiome will be resilient and overcome the disturbance [43], supporting the overall holobiont homeostasis.

## Conclusions

Understanding the effect of environmental fluctuation and stress on the biotic community is a new challenge to overcome to produce reliable prediction models aim to evaluate the possible consequences that climate change may have on Earth’s biomes. Therefore, it is increasingly important to measure the biotic response to environmental variability/fluctuations over time. With this study we report the capability of a perennial desert plant, exposed to seasonal environmental fluctuations, to determine stable conditions that select for a consistent bacterial community across two contrasting seasons (winter *vs*. summer). This demonstrates the capability of plants in desert ecosystems to homogenize and stabilize their microbial community, minimizing the heterogeneity of the surrounding ecosystem (bulk sand) through a combination of deterministic and stochastic processes. The results of this study set a research framework for implementing further log-term studies that encompass multiple seasons and intermediate assessments between/within seasons to elucidate the microbial community dynamic at finer, longer and ecologically relevant time scales.

Since the rhizosheath is an effective adaptation of speargrasses to drought stress, and also occurs in several economically important crops (such as cereals), here we highlight how the stabilization of the plant-microbiome represents an important mechanism to buffer environmental changes that could be further explored as a potential sustainable agricultural practice.

## Supplementary Information

**Additional file 1. Result S1**. Analysis of rhizosheath sand composition. Result S2. Analysis of cultivable bacteria. **Table S1**. Climatic conditions throughout the year in Ksar Ghilane, Sahara Desert (Tunisia); data elaborated from https://www.worldweatheronline.com; data as reported as monthly average measurement from January 2019 to now. **Table S2**. List of FISH probes used and conditions applied; probe sequence (5’-3’), attached fluorochrome (fluor.), bacterial target group, percentage of formaldehyde (FA) used during treatment, and references are also reported. **Table S3**. Number of KTUs and sequences used for each sample. RH: rhizosphere, RS: rhizosheath, RT: root tissue, BS: bulk sand. **Table S4**. Humidity (RH%) and temperature (T°C) measured in summer (June, 2016) and winter (November, 2016) for bulk sand and belowground speargrasses (*i.e.*, plant rhizosheath–root system, RS). **Table S5**. Physico-chemical analyses conducted on bulk sand (BS) and rhizosheath matrix (RS) collected in summer (S) and winter (W). **Table S6**. PERMANOVA pair-wise comparison test of physico-chemical conditions (Table S5) in rhizosheath (RS) and bulk sand (BS) collected during summer (S) and winter (W) seasons. **Table S7**. Multivariate test (pairwise comparison) of beta-diversity associated with root tissue, rhizosheath, rhizosphere, and bulk sand. **Table S8**. Mean and standard error of multivariate dispersions from centroid calculated for each compartment (within-betadiversity). **Table S9**. Relative importance of different ecological processes in the assembly of bacterial community associated with the rhizosheath-root system compartments and bulk sand in two contrasting seasons. **Table S10**. Generalized linear model univariate test indicates the KTUs contributing to the difference in bulk soil among summer and winter. **Table S11**. Tukey’s honest significance difference (TukeyHSD) pairwise comparison tests for the degree, betweenness and keystone species detected across the four co-occurrence networks, namely bulk sand winter, bulk sand winter summer, rhizosheath–root system winter and rhizosheath–root system summer. **Table S12**. List of bacterial isolates, PGP activity, and abiotic resistance tested *in vitro*. **Figure S1**. *Stipagrostis pungens* rhizosheath-root system. **Figure S2**. Rarefaction curves of bacterial reads obtained by pair-ends MiSeq Illumina sequencing in bulk sand, rhizosheath, rhizosphere, and root tissues. **Figure S3**. Visualization of *Stipagrostis pungens* rhizosheath with scanning electron microscopy (SEM). **Figure S4**. SEM images and electron micrographs of the sand grains and root tissue within the rhizosheath of *Stipagrostis pungens *using environmental scanning electron microscopy (ESEM) to reveal chemical composition. **Figure S5**. Localization of bacteria in rhizosheath-root system by confocal laser-scanning microscopy (CLSM) and fluorescence *in situ* hybridization (FISH). **Figure S6**. Bacterial KTUs distribution across samples. **Figure S7**. Venn diagram shows the distribution of bacterial KTUs across compartment categories. **Figure S8**. Alpha diversity expressed as richness (number of KTUs) and Shannon diversity across the compartment categories. **Figure S9**. Quantification of betadiversity components in bulk sand bacterial communities across seasons (summer and winter). **Figure S10**. Alpha diversity expressed as richness (number of KTUs) and Shannon diversity across the compartments in summer and winter. **Figure S11**. Venn diagrams showing the number of KTUs present in summer and/or in winter in each compartment category. **Figure S12**. Analysis of 2-fold change was performed to evaluate the KTUs that had a significantly (*p* < 0.01) different relative abundance (2-fold change) over summer and winter. **Figure S13**. Taxonomy of co-occurrence network degrees in bulk sand and rhizosheath matrix across seasons. **Figure S14**. Microcosms to evaluate sand wettability *in vitro*. Evaluation of sand weight at 48 h and along the entire incubation (0, 24 and 48 h).

**Additional file 2. Data S1.** Bacterial KTUs table with taxonomic information. Refer to excel file: Marasco et al 2021_Supplementary Data S1.

## Data Availability

The datasets analysed during the current study are available in the NCBI SRA repository under the BioProject ID PRJNA745579 and SUB9992284.

## Abbreviations

rRNA: Ribosomal ribonucleic acid
EPS: Extracellular polymeric substances
PGP: Plant growth promoting
DNA: Deoxyribonucleic acid
TSA: Tryptone soy agar
ANOVA: Analysis of variance
C: Carbon
N: Nitrogen
PERMANOVA: Permutational multivariate analysis of variance
SIMPER: Analysis of similarity percentages
PRIMER: Plymouth routines in multivariate ecological research
SEM: Scanning electron microscope
FISH: Fluorescent in situ hybridization
CLSM: Confocal laser scanning microscopy
PBS: Phosphate-buffered saline
TC: Total carbohydrates
W-EPS: Water-extractable EPS
EDTA: Ethylenediaminetetraacetic acid
E-EPS: EDTA-extractable EPS
IEC: Ion-exchange chromatography
MW: Molecular weight
PCR: Polymerase chain reaction
QIIME: Quantitative Insights Into Microbial Ecology
KTU: Operational taxonomic unit
BC: Bray–Curtis
PCoA: Principal coordinate analysis
KLD: Kullback-Leiber
R2A: Reasoner's 2A
CFUs: Colony-forming units
ITS: Internal transcribed spacer
IAA: Indole acetic acid
PEG: Polyethylene glycol
WC: Water content
RWC: Relative water content
DW: Dry weight
GLM: Generalized multivariate linear model
PERMDISP: Permutational analysis of multivariate dispersions
HSD: Honestly significant difference
NC: Negative control
