# Supplementary material for: Rhizosheath–root system changes exopolysaccharide content but stabilizes bacterial community across contrasting seasons in a desert environment
Source: Environ Microbiome. 2022 Apr 1;17:14. doi: 10.1186/s40793-022-00407-3 (PMC8973986; doi:10.1186/s40793-022-00407-3)
Supplement: Supplementary file 1 — Additional file 1. Result S1. Analysis of rhizosheath sand composition. Result S2. Analysis of cultivable bacteria. Table S1. Climatic conditions throughout the year in Ksar Ghilane, Sahara Desert (Tunisia); data elaborated from https://www.worldweatheronline.com; data as reported as monthly average measurement from January 2019 to now. Table S2. List of FISH probes used and conditions applied; probe sequence (5’-3’), attached fluorochrome (fluor.), bacterial target group, percentage of formaldehyde (FA) used during treatment, and references are also reported. Table S3. Number of KTUs and sequences used for each sample. RH: rhizosphere, RS: rhizosheath, RT: root tissue, BS: bulk sand. Table S4. Humidity (RH%) and temperature (T°C) measured in summer (June, 2016) and winter (November, 2016) for bulk sand and belowground speargrasses (i.e., plant rhizosheath–root system, RS). Table S5. Physico-chemical analyses conducted on bulk sand (BS) and rhizosheath matrix (RS) collected in summer (S) and winter (W). Table S6. PERMANOVA pair-wise comparison test of physico-chemical conditions (Table S5) in rhizosheath (RS) and bulk sand (BS) collected during summer (S) and winter (W) seasons. Table S7. Multivariate test (pairwise comparison) of beta-diversity associated with root tissue, rhizosheath, rhizosphere, and bulk sand. Table S8. Mean and standard error of multivariate dispersions from centroid calculated for each compartment (within-betadiversity). Table S9. Relative importance of different ecological processes in the assembly of bacterial community associated with the rhizosheath-root system compartments and bulk sand in two contrasting seasons. Table S10. Generalized linear model univariate test indicates the KTUs contributing to the difference in bulk soil among summer and winter. Table S11. Tukey’s honest significance difference (TukeyHSD) pairwise comparison tests for the degree, betweenness and keystone species detected across the four co-occurrence networks, namel [file 40793_2022_407_MOESM1_ESM.pdf]

Additional file

Rhizosheath–root system changes exopolysaccharide content but stabilizes bacterial community across contrasting seasons in a desert environment

Ramona Marasco[1]*, Marco Fusi[1,‡], Maria Mosqueira[1], Jenny Booth[1], Federico Rossi[2], Massimiliano Cardinale[3,4], Grégoire Michoud[1], Eleonora Rolli[5], Gianmarco Mugnai[6], Lorenzo Vergani[5], Sara Borin[5], Roberto De Philippis[2,6], Ameur Cherif[7], and Daniele Daffonchio[1]*

[1]Biological and Environmental Sciences and Engineering Division (BESE), King Abdullah University of Science and Technology (KAUST), Thuwal 23955-6900, Kingdom of Saudi Arabia
[2]Department of Agriculture, Food, Environment & Forestry (DAGRI), University of Florence, Florence, Italy
[3]Department of Biological and Environmental Sciences and Technologies, University of Salento, Lecce, Italy,
[4]Institute of Applied Microbiology, Research Center for BioSystems, Land Use, and Nutrition (IFZ), Justus-Liebig-University, Giessen, Germany
[5]Department of Food, Environmental and Nutritional Sciences (DeFENS), University of Milano, Milan, Italy
[6]Institute of BioEconomy, CNR, Sesto Fiorentino, Florence, Italy
[7]Institut Supérieur de Biotechnologie Sidi Thabet (ISBST), BVBGR-LR11ES31, Biotechpole Sidi Thabet, University Manouba, Ariana, Tunisia

*Correspondence to Ramona Marasco, ramona.marasco@kaust.edu.sa and Daniele Daffonchio, daniele.daffonchio@kaust.edu.sa

[‡]Present Address: Joint Nature Conservation Committee, Monkstone House, City Road, Peterborough, PE1 1JY, UK

Supplementary Data

Supplementary Data S1. Bacterial KTUs table with taxonomic information. Refer to excel file: Marasco et al 2021_Supplementary Data S1.

Supplementary Results

Supplementary Result S1. Analysis of rhizosheath sand composition. We detected changes in size of sand trapped in the rhizosheath compared to the dune sand. Both types of samples were dominated by fine sand (100–250 µm; bulk sand = 79.3 ± 0.9 and rhizosheath = 75.0 ± 1.9) and very fine sand (<100 µm; bulk sand = 20.7.3 ± 0.9 and rhizosheath = 25.0 ± 1.9); the fine sand showed higher percentage in rhizosheath than in bulk sand in both seasons (Tukey's multiple comparisons test: winter, $p = 0.013$ and summer, $p = 0.025$;); medium and coarse sands (>250 µm) were not detected.

Supplementary Result S2. Analysis of cultivable bacteria. The number of cultivable bacteria (CFU/g sample) in the three oligotrophic media (R2A, R2A diluted, and R2A + 5% NaCl) ranged between 1×10^6 to 2×10^7 CFU/g of root tissues, and between 4×10^6 to 4×10^8 CFU/g of rhizosheath matrix. The bacteria isolated (n = 289; 140 and 149 from the root tissues and rhizosheath matrix) belong to 21 genera; the most abundant genera in the root tissues were *Rathayibacter* (66 of isolates), *Exiguobacterium* (39), *Bacillus* (5) *Stenotrophomonas* (5) and *Xanthomonas* (5), while in the rhizosheath matrix were *Streptomyces* (90), *Bacillus* (19), *Brachybacterium* (10), *Staphylococcus* (10), *Enterococcus* (6) and *Enterobacter* (5).

Supplementary Tables

Supplementary Table S1. Climatic conditions throughout the year in Ksar Ghilane, Sahara Desert (Tunisia); data elaborated from https://www.worldweatheronline.com; data as reported as monthly average measurement from January 2019 to now.

Climatic Condition	January	February	March	April	May	June	July	August	September	October	November	December
T max (°C)	15	17	20	24	28	32	35	35	31	27	20	16
T min (°C)	8	9	11	15	19	22	24	24	23	19	13	9
Precipitation (mm)	14.6	20.7	29.1	14.5	19.7	9.9	4.3	9.1	18.5	27.6	40	17.6
Rainfall days (n)	5	5	8	6	6	3	2	2	6	7	6	4
Air humidity (%)	48	42	36	48	41	41	38	43	51	53	50	66
Pressure (mb)	1019	1017	1016	1013	1014	1013	1013	1014	1015	1016	1016	1021

Supplementary Table S2. List of FISH probes used and conditions applied; probe sequence (5'-3'), attached fluorochrome (fluor.), bacterial target group, percentage of formaldehyde (FA) used during treatment, and references are also reported.

Name	Sequence (5'→3')	Fluor.	Target group	%FA#	Reference
EUB338*	GCTGCCTCCCGTAGGAGT	Cy3	Most bacteria	10	[1]
EUB338II*	GCAGCCACCCGTAGGTGT	Cy3	*Planctomycetales*	10	[2]
EUB338III*	GCTGCCACCCGTAGGTGT	Cy3	*Verrucomicrobiales*	10	[2]
ALF968	GGTAAGGTTCTGCGCGTT	Cy5	*Alphaproteobacteria*	35	[3]
BET42a	GCCTTCCCACTTCGTTT	FITC	*Betaproteobateria*	35	[4]
GAM42a	GCCTTCCCACATCGTTT	Cy5	*Gammaproteobacteria*	35	[4]
HGC236	AACAAGCTGATAGGCCGC	Cy5	*Actinobacteria*	10	[5]
NONEUB-Cy5	ACTCCTACGGGAGGCAGC	Cy5	/	***	
NONEUB-Cy3	ACTCCTACGGGAGGCAGC	Cy3	/	***	[6]
NONEUB-FITC	ACTCCTACGGGAGGCAGC	FITC	/	***	

*Applied as an equimolar mix
***The same stringency conditions of the FISH probe with the same fluorescent
#Incubation at 40°C

Supplementary Table S3. Number of KTUs and sequences used for each sample. RH: rhizosphere, RS: rhizosheath, RT: root tissue, BS: bulk sand.

Id	Season	Fraction	N. KTUs	Used	Plastid	Archaea	Unassigned	Good's value
D01	Summer	RH	506	41744	0	0	0	99.998
D02	Summer	RH	444	35376	3	0	0	99.997
D03	Summer	RH	586	77399	28	6	0	99.996
D04	Summer	RH	520	70888	12	0	0	99.997
D05	Summer	RH	548	86616	105	0	0	99.995
D06	Summer	RH	653	64026	3	17	0	99.997
D07	Summer	RH	590	54144	13	0	0	99.994
D08	Summer	RS	330	43895	2325	0	0	99.995
D09	Summer	RS	310	43419	381	0	0	100.000
D10	Summer	RS	373	46898	760	0	0	99.991
D11	Summer	RS	326	33539	1773	0	0	100.000
D12	Summer	RS	305	30513	468	0	0	100.000
D13	Summer	RS	313	25671	61	0	0	99.992
D14	Summer	RS	322	25987	225	0	0	99.996
D15	Summer	RT	17	10024	9189	0	0	100.000
D16	Summer	RT	9	28342	46983	0	0	99.996
D17	Summer	RT	5	16819	53505	0	0	100.000
D18	Summer	RT	7	13735	55802	0	0	100.000
D19	Summer	RT	3	15053	45888	0	0	100.000
D20	Summer	RT	4	29904	41060	0	0	100.000
D21	Summer	RT	11	17598	40292	0	0	100.000
D22	Summer	BS	806	82202	0	95	0	100.000
D23	Summer	BS	689	85451	0	25	0	100.000
D24	Summer	BS	370	19443	0	10	0	100.000
D25	Summer	BS	258	22315	0	28	0	100.000
D26	Summer	BS	512	49325	36	28	0	100.000
D27	Summer	BS	354	29696	0	13	0	100.000
D28	Summer	BS	152	9916	0	0	0	99.990
G01	Winter	RH	398	32099	26	0	0	100.000
G02	Winter	RH	280	29245	21	0	0	100.000
G03	Winter	RH	496	45636	0	2	0	99.996
G04	Winter	RH	521	45273	6	0	0	99.991
G05	Winter	RH	490	43280	18	3	0	99.988
G06	Winter	RH	602	62090	25	0	0	99.992
G07	Winter	RH	542	49172	55	8	0	99.988
G08	Winter	RS	416	46442	816	0	0	99.996
G09	Winter	RS	416	53270	539	0	0	100.000
G10	Winter	RS	480	64733	1004	0	0	99.997
G11	Winter	RS	404	63293	1232	0	0	100.000
G12	Winter	RS	130	25416	1402	0	0	100.000
G13	Winter	RS	474	55486	2136	2	0	100.000
G14	Winter	RS	502	63760	1226	0	0	99.995
G15	Winter	RT	3	13126	43134	0	0	100.000
G16	Winter	RT	28	9726	24916	0	0	100.000
G17	Winter	RT	21	10479	26360	0	0	100.000
G18	Winter	RT	5	12269	94163	0	0	100.000
G19	Winter	RT	4	12265	36767	0	0	100.000
G20	Winter	RT	5	11593	46995	0	0	100.000
G21	Winter	RT	3	60043	5902	0	0	100.000
G22	Winter	BS	1335	228897	190	6	0	100.000
G23	Winter	BS	1360	239889	232	45	0	99.999
G24	Winter	BS	1321	262409	199	86	0	99.998
G25	Winter	BS	1155	203460	58	92	33	100.000
G26	Winter	BS	1319	216077	61	0	0	100.000
G27	Winter	BS	997	88378	0	44	0	99.998
G28	Winter	BS	1357	181622	65	41	6	100.000

Supplementary Table S4. Humidity (RH%) and temperature (T°C) measured in summer (June, 2016) and winter (November, 2016) for bulk sand and belowground speargrasses (*i.e.*, plant rhizosheath–root system, RS); measurements were collected on three consecutive days at 6:00, 12:00, 18:00 and 0:00 in three different locations; bulk sand and plants RS at 5 cm (surface) and 20 cm (deep) were considered. Air conditions were also registered; asterisk (*) indicates data from https://www.worldweatheronline.com, not directly measured in the field. N.d., not determined.

	Time		Air	Surface		Deep (20 cm)	
	**Month**	**Hour**		**Bulk sand**	**Plant RS**	**Bulk sand**	**Plant RS**
RH%	Jun, 2016	6	41*	2.3 ± 0.55	3.3 ± 0.63	10.5 ± 3.51	13.5 ± 2.55
		**12**		**1.52 ± 0.25**	**3.12 ± 0.34**	**2.75 ± 0.30**	**6.90 ± 0.43**
		18		1.19 ± 0.14	2.68 ± 0.24	16.88 ± 5.60	17.71 ± .78
		24		2.5 ± 0.35	n.d.	17.05 ± 2.61	n.d.
	Nov, 2016	6	48*	54.8 ± 0.9	65.8 ± 3.6	51.8 ± 0.1	64.4 ± 15.3
		**12**		**36.7 ± 1.3**	**40.0 ± 3.5**	**37.1 ± 1.7**	**43.9 ± 3.2**
		18		31.2 ± 1.1	64.6 ± 2.5	52.2 ± 0.1	70.6 ± 5.4
		24		51.7 ± 0.5	65.6 ± 2.9	52.2 ± 0.1	65.3 ± 13.8
T°C	Jun, 2016	6	24.3 ± 1.0	20.3 ± 0.5	22.3 ± 0.2	27.1 ± 0.2	27.3 ± 0.1
		**12**	**41.2 ± 0.8**	**47.4 ± 1.1**	**38.1 ± 2.3**	**32.2 ± 1.9**	**28.5 ± 0.9**
		18	38.5 ± 0.5	46.5 ± 0.8	37.1 ± 1.0	29.9 ± 0.5	29.1 ± 0.8
		24	27.0 ± 1.0	29.7 ± 0.2	n.d.	28.7 ± 0.9	n.d.
	Nov, 2016	6	15.5 ± 0.5	6.7 ± 0.3	22.2 ± 1.1	19.8 ± 1.8	20.8 ± 0.5
		**12**	21.5 ± 1.0	**16.0 ± 1.1**	**20.8 ± 0.1**	**16.7 ± 0.8**	**20.0 ± 0.1**
		18	18.0 ± 0.6	18.7 ± 0.1	21.5 ± 0.2	17.7 ± 1.1	20.5 ± 0.2
		24	16.0 ± 0.5	9.6 ± 0.2	21.9 ± 0.3	19.4 ± 0.3	20.7 ± 0.3

Supplementary Table S5. Physico-chemical analyses conducted on bulk sand (BS) and rhizosheath matrix (RS) collected in summer (S) and winter (W); values are expressed as mean ± standard deviation of three replicates. Analyses were conducted at the GEOMAR laboratory (Germany). Asterisk (*) indicates significance of ANOVA (*p*-value < 0 .05) and lower-case letters indicate significant differences ($p < 0.05$) between the four sample categories: BS-S, bulk sand summer; BSW, bulk sand winter; RS-S, rhizosheath summer; RS-W, rhizosheath winter.

**Parameter**	**BS-S**	**BS-W**	**RS-S**	**RS-W**
pH*	7.29 ± 0.03 (ab)	7.34 ± 0.185 (a)	7.033 ± 0.032 (b)	7.073 ± 0.068 (b)
Total C*	0.601 ± 0.016 (a)	0.611 ± 0.1 (a)	0.832 ± 0.008 (b)	0.82 ± 0.04 (b)
Organic C *	0.075 ± 0.023 (a)	0.098 ± 0.027 (a)	0.609 ± 0.103 (b)	0.427 ± 0.071 (c)
Inorganic C *	0.525 ± 0.036 (a)	0.513 ± 0.1 (ab)	0.223 ± 0.111 (b)	0.393 ± 0.099 (b)
Total N	0.479 ± 0.015 (ab)	0.526 ± 0.063 (a)	0.327 ± 0.016 (c)	0.394 ± 0.07 (bc)
Organic N*	0.063 ± 0.004 (a)	0.064 ± 0.004 (ab)	0.076 ± 0.003 (cb)	0.077 ± 0.007 (c)
Inorganic N*	0.416 ± 0.017 (ab)	0.461 ± 0.063 (a)	0.25 ± 0.013 (c)	0.316 ± 0.068 (bc)
Nitrite	0.00005 ± 0.000004	0.0007 ± 0.000009	0.00005 ± 0.000007	0.00005 ± 0.00002
Nitrate*	0.0027 ± 0.00014 (a)	0.003 ± 0.00042 (a)	0.0012 ± 0.00014 (b)	0.0011 ± 0.00013 (b)
Phosphate	0.0019 ± 0.00035	0.0021 ± 0.00092	0.0011 ± 0.0003	0.0016 ± 0.00011
Silicate*	0.0074 ± 0.00063 (a)	0.0067 ± 0.00025 (a)	0.0074 ± 0.00019 (a)	0.0055 ± 0.00036 (b)

Supplementary Table S6. PERMANOVA pairwise comparison test of physico-chemical conditions (Supplementary Table S5) in rhizosheath (RS) and bulk sand (BS) collected during summer (S) and winter (W) seasons.

**Groups**	**t**	***p***
BS-S, BS-W	1.0378	0.386
BS-S, RS-S	6.7552	0.002
BS-S, RS-W	3.6758	0.006
BS-W, RS-S	5.5618	0.002
BS-W, RS-W	3.0873	0.009
RS-S, RS-W	2.1603	0.056

Supplementary Table S7. Multivariate test (pairwise comparison) of betadiversity associated with root tissue, rhizosheath, rhizosphere, and bulk sand.

**Groups**	**Dev**	***p***
Rhizosphere, Rhizosheath	2993	0.003
Rhizosphere, Root tissue	16474	0.001
Rhizosphere, Bulk sand	13138	0.001
Rhizosheath, Root tissue	10817	0.001
Rhizosheath, Bulk sand	17262	0.001
Root tissue, Bulk sand	26788	0.001

Supplementary Table S8. Mean and standard error of multivariate dispersions from centroid calculated for each compartment (within-betadiversity).

**Group**	**Replicates**	**Average**	**St.Er.**
Rhizosphere	14	30.674	1.658
Rhizosheath	14	31.835	1.372
Root tissue	14	44.308	3.103
Bulk sand	14	36.193	3.277

Supplementary Table S9. Relative importance of different ecological processes in the assembly of bacterial community associated with the rhizosheath-root system compartments and bulk sand in two contrasting seasons. Data obtained by iCAMP are presented as mean values ± standard deviation; L, M, S, and N represented large (|d| > 0.8), medium (0.5 < |d| ≤ 0.8), small (0.2 < |d| ≤ 0.5), and negligible (|d| ≤ 0.2) effect sizes of season in the four compartments (comp.; BS, bulk sand; RH, rhizosphere; RS, rhizosheath; RT, root tissue) based on Cohen's D tests; differences of the process between the two seasons are also reported (significance, *p*-values < 0.05).

Comp.	Season/Cohen's D test/p-value	Heterogeneous selection	Homogeneous selection	Dispersal limitation	Homogenizing dispersal	Drift and others
**BS**	Summer	0.001 ± 0.001	0.14 ± 0.014	0.057 ± 0.023	0.136 ± 0.046	0.667 ± 0.06
	Winter	0.001 ± 0.001	0.432 ± 0.024	0.169 ± 0.045	0.026 ± 0.03	0.373 ± 0.035
	Cohen's D	N	L	L	L	L
	p-value	0.57	< 0. 0001	0.015	0.037	< 0.0001
**RH**	Summer	0	0.126 ± 0.021	0.334 ± 0.054	0.001 ± 0.0005	0.539 ± 0.054
	Winter	0.001 ± 0.001	0.09 ± 0.016	0.309 ± 0.076	0.004 ± 0.005	0.597 ± 0.078
	Cohen's D	L	L	S	L	L
	p-value	0.116	0.074	0.388	0.116	0.268
**RS**	Summer	0.052 ± 0.01	0.245 ± 0.051	0.256 ± 0.085	0.002 ± 0.002	0.444 ± 0.091
	Winter	0.041 ± 0.012	0.26 ± 0.055	0.192 ± 0.079	0.043 ± 0.041	0.464 ± 0.082
	Cohen's D	L	S	M	L	S
	p-value	0.216	0.413	0.287	0.339	0.433
**RT**	Summer	0.123 ± 0.015	0.725 ± 0.048	0.073 ± 0.027	0	0.079 ± 0.022
	Winter	0.215 ± 0.135	0.598 ± 0.154	0.097 ± 0.095	0	0.09 ± 0.058
	Cohen's D	L	L	L		M
	p-value	0.345	0.071	0.086		0.319

Supplementary Table S10. Generalized linear model univariate test indicates the KTUs contributing to the difference in bulk soil among summer and winter; data are reported at class level; high values of deviance (dev.) correspond to high contributions to diversity. Only KTUs with deviance > 20 are showed.

KTU_ID	Dev.	*p*-value	Class	KTU_ID	Dev.	*p*-value	Class
KTU_1225	44.71	0.002	*Bacilli*	KTU_3255	31.65	0.003	*Oligoflexia*
KTU_0944	44.67	0.002	*Anaerolineae*	KTU_1251	31.55	0.003	*Alphaproteobacteria*
KTU_0510	43.46	0.003	*Anaerolineae*	KTU_3057	31.42	0.002	*Chloroflexia*
KTU_0070	42.68	0.003	*Chloroflexia*	KTU_1501	31.21	0.002	*Verrucomicrobiae*
KTU_0471	41.39	0.003	*Chloroflexia*	KTU_0540	30.79	0.003	*Verrucomicrobiae*
KTU_0559	41.02	0.003	*Planctomycetes*	KTU_0842	30.73	0.002	*Alphaproteobacteria*
KTU_0114	40.62	0.003	*Bacteroidia*	KTU_1697	30.49	0.003	*Phycisphaerae*
KTU_0451	39.80	0.002	*Chloroflexia*	KTU_0576	30.38	0.003	*Gammaproteobacteria*
KTU_2951	39.79	0.003	*Planctomycetes*	KTU_2535	30.29	0.003	*Bacilli*
KTU_0926	39.17	0.003	*Acidobacteriae*	KTU_2612	29.56	0.002	*Chloroflexia*
KTU_2761	38.68	0.002	*Chloroflexia*	KTU_2945	29.47	0.003	*Acidimicrobiia*
KTU_0824	38.24	0.002	*Verrucomicrobiae*	KTU_2640	29.46	0.002	*Thermoleophilia*
KTU_2362	37.37	0.003	*Saccharimonadia*	KTU_0130	28.87	0.003	*Chloroflexia*
KTU_0261	37.03	0.002	*Verrucomicrobiae*	KTU_2862	28.84	0.003	*Polyangia*
KTU_0232	36.96	0.002	*Alphaproteobacteria*	KTU_2655	28.83	0.003	*Bacteroidia*
KTU_0295	36.30	0.003	*Verrucomicrobiae*	KTU_1169	28.82	0.003	*Verrucomicrobiae*
KTU_0595	36.23	0.002	*Planctomycetes*	KTU_1611	28.78	0.003	*Anaerolineae*
KTU_2285	35.75	0.003	*Chloroflexia*	KTU_2584	28.06	0.002	*Verrucomicrobiae*
KTU_0361	35.73	0.002	*Verrucomicrobiae*	KTU_0271	28.00	0.002	*Bacteroidia*
KTU_0479	35.68	0.003	*Chloroflexia*	KTU_0421	27.77	0.003	*Bacteroidia*
KTU_0351	35.27	0.003	*Verrucomicrobiae*	KTU_0985	27.30	0.003	*Gammaproteobacteria*
KTU_0333	34.97	0.003	*Bacilli*	KTU_0550	27.25	0.003	*Verrucomicrobiae*
KTU_2696	34.53	0.002	*Verrucomicrobiae*	KTU_2411	26.97	0.002	*Bdellovibrionia*
KTU_0511	34.45	0.003	*Phycisphaerae*	KTU_0714	26.90	0.002	*Bacteroidia*
KTU_2114	34.39	0.002	*Alphaproteobacteria*	KTU_1665	26.65	0.002	*Bdellovibrionia*
KTU_2714	34.19	0.002	*Blastocatellia*	KTU_0530	26.48	0.002	*Gammaproteobacteria*
KTU_2868	33.94	0.003	*Longimicrobia*	KTU_2668	26.36	0.003	*Symbiobacteriia*
KTU_2579	33.89	0.003	*Bdellovibrionia*	KTU_0883	26.34	0.002	*Polyangia*
KTU_0431	33.82	0.003	*Alphaproteobacteria*	KTU_2977	26.24	0.003	*Alphaproteobacteria*
KTU_2627	33.77	0.003	*Acidobacteriae*	KTU_2863	26.19	0.002	*Bacteroidia*
KTU_0536	33.70	0.002	*Thermoleophilia*	KTU_2685	25.72	0.002	*Bdellovibrionia*
KTU_3129	33.69	0.002	*Sumerlaeia*	KTU_0010	25.60	0.002	*Bacilli*
KTU_0456	33.40	0.002	*Polyangia*	KTU_1121	25.58	0.003	*Bacteroidia*
KTU_0053	32.92	0.002	*Nitrospiria*	KTU_2590	25.54	0.002	*Bacilli*
KTU_0498	32.85	0.003	*Verrucomicrobiae*	KTU_0397	25.29	0.002	*Bacteroidia*
KTU_2692	32.72	0.002	*Chloroflexia*	KTU_2595	24.67	0.003	*Verrucomicrobiae*
KTU_0291	32.59	0.002	*Verrucomicrobiae*	KTU_1018	23.88	0.003	*Bacteroidia*
KTU_2697	32.45	0.002	*Chloroflexia*	KTU_2824	23.72	0.002	WPS-*2*
KTU_0871	32.38	0.003	*Alphaproteobacteria*	KTU_0044	22.88	0.003	*Bacteroidia*
KTU_2804	32.23	0.002	Armatimonadota unc.	KTU_2537	22.38	0.003	*Thermoleophilia*
KTU_1295	32.00	0.003	*Verrucomicrobiae*	KTU_2682	21.47	0.003	*Dojkabacteria*
KTU_2693	31.68	0.002	*Chloroflexia*	KTU_0043	21.27	0.003	*Gammaproteobacteria*

Supplementary Table S11. Tukey's honest significance difference (TukeyHSD) pariwise comparison tests for the degree, betweenness and keystone species detected across the four co-occurrence networks, namely bulk sand winter, bulk sand winter summer, rhizosheath–root system winter and rhizosheath–root system summer; BS, bulk sand and RS, rhizosheath–root system. Mean differences and *p*-values are reported; significance, *p*-value < 0.05.

**Network parameter**	**Comparison**	**Mean difference**	***p*-value**
Degree	BSWinter-BSSummer	-0.51845605	<0.0001
	RSSummer-BSSummer	-0.22461307	0.0000061
	RSWinter-BSSummer	-0.18477735	0.0002929
	RSSummer-BSWinter	0.29384298	<0.0001
	RSWinter-BSWinter	0.33367869	<0.0001
	RSWinter-RSSummer	0.03983571	0.8267534
Betweenness	BSWinter-BSSummer	-2104.5994	0.0029018
	RSSummer-BSSummer	-2506.7808	0.0007137
	RSWinter-BSSummer	-2319.2433	0.0019483
	RSSummer-BSWinter	-402.1814	0.9161048
	RSWinter-BSWinter	-214.6439	0.9853982
	RSWinter-RSSummer	187.5375	0.9920304
Keystone species	BSWinter-BSSummer	-19.778827	0.0078001
	RSSummer-BSSummer	-14.549399	0.0800486
	RSWinter-BSSummer	-13.537363	0.116537
	RSSummer-BSWinter	5.229428	0.8186083
	RSWinter-BSWinter	6.241464	0.7251912
	RSWinter-RSSummer	1.012037	0.9982683

Supplementary Table S12. List of bacterial isolates, PGP activity, and abiotic resistance tested *in vitro*.

Strain	N. isolates*	Closest relative	EPS	Sol P	SID	IAA	4 °C	37 °C	42 °C	50 °C	5%NaCl	8%NaCl	20%PEG
E001	3	*Stenotrophomonas maltophilia*			+		+	+				+	+
E002	1	*Bacillus simplex*	+			+	+	+	+		+	+	+
E003	1	*Rathayibacter tritici*				+		+	+				+
E004	1	*Rathayibacter tritici*				+		+	+				+
E011	1	*Paenibacillus ginsengagri*				+		+	+				
E014	1	*Acinetobacter lwofii*						+	+				+
E015	2	*Rathayibacter tanaceti*				+		+	+				+
E016	7	*Rathayibacter tanaceti*				+		+	+				+
E019	1	*Brevibacillus limnophilus*				+		+	+	+			
E021	8	*Rathayibacter tritici*				+		+	+				+
E024	1	*Exiguobacterium mexicanum*				+		+					+
E025	1	*Moraxella osloensis*				+		+					
E026	1	*Acinetobacter lwoffii*						+	+				+
E028	2	*Acinetobacter lwoffii*						+	+				+
E031	1	*Acinetobacter lwoffii*						+	+				+
E033	4	*Xanthomonas arboricola*			+		+	+				+	+
E034	4	*Bacillus simplex*	+			+	+	+	+				+
E035	1	*Exiguobacterium homiense*						+	+				
E042	2	*Geobacillus thermoleovorans*				+	+	+	+		+		+
E045	1	*Paenibacillus xylanexedens*						+					+
E046	1	*Geobacillus bogazici*						+	+			+	
E054	37	*Exiguobacterium mexicanum*						+	+				
E062	2	*Staphylococcus capitis*						+	+		+		+
E076	1	*Brevibacillus borstelensis*				+		+	+	+			+
E084	1	*Micrococcus luteus*				+		+	+		+		+
E098	2	*Kocuria rizhophila*						+	+		+		+
E099	1	*Micrococcus yunnanensis*				+		+	+		+		+
E106	5	*Rathayibacter tritici*				+	+	+	+				+
E108	39	*Rathayibacter tritici*				+		+	+				+
E128	2	*Stenotrophomonas maltophilia*			+		+	+				+	
E137	1	*Xanthomonas arboricola*	+			+		+	+				+
E141	1	*Rathayibacter tritici*				+	+	+	+				+
E147	1	*Bosea minatitlanensis*				+		+	+				+
E149	1	*Rathayibacter tritici*				+	+	+	+				+
E154	1	*Rathayibacter tritici*				+	+	+	+				+

Strain	N. isolates*	Closest relative	EPS	Sol P	Sid	IAA	4 °C	37 °C	42 °C	50 °C	5%NaCl	8%NaCl	20%PEG
R001	3	*Streptomyces ambofaciens*						+	+				+
R005	4	*Streptomyces albogriseolus*	+	+		+		+	+		+	+	+
R006	1	*Paenibacillus lautus*	+			+		+	+				+
R010	1	*Streptomyces mutabilis*	+	+		+		+	+		+	+	+
R012	1	*Enterobacter hormaechei*	+			+	+	+	+		+	+	+
R014	1	*Streptomyces atrovirens*	+			+		+	+				+
R016	1	*Streptomyces albogriseolus*	+	+		+		+	+		+		+
R017	1	*Pseudomonas putida*				+	+	+	+				+
R019	1	*Bacillus simplex*	+			+	+	+	+				+
R022	1	*Streptomyces mutabilis*	+	+		+		+	+		+	+	+
R024	10	*Streptomyces mutabilis*	+			+		+	+		+		+
R026	1	*Streptomyces mutabilis*	+				+	+		+	+	+	+
R028	1	*Streptomyces atrovirens*	+		+		+	+				+	+
R032	1	*Streptomyces paradoxus*	+		+	+	+	+				+	+
R034	3	*Brevibacillus halotolerans*					+	+				+	+
R037	4	*Bacillus zanthoxyli*			+	+	+	+		+		+	+
R049	2	*Staphylococcus warneri*	+			+	+	+		+	+	+	+
R051	1	*Streptomyces lavendulocolor*					+	+				+	+
R052	1	*Streptomyces mutabilis*				+		+	+		+	+	+
R053	1	*Enterobacter cancerogenus*	+	+		+	+	+	+		+		+
R054	6	*Bacillus zanthoxyli*	+	+		+		+	+		+	+	+
R055	1	*Staphylococcus succinus*	+	+		+		+	+		+	+	+
R056	1	*Bacillus licheniformis*				+		+	+	+	+	+	+
R057	2	*Microbacterium indicum*	+	+		+	+	+	+		+	+	+
R059	3	*Streptomyces mutabilis*	+	+		+		+			+		+
R064	7	*Staphylococcus succinus*	+			+	+	+	+		+	+	+
R065	3	*Streptomyces mutabilis*				+		+	+		+	+	+
R069	1	*Microbacterium sorbitolivorans*	+			+	+	+	+		+		+
R071	1	*Bacillus tropicus*		+		+		+	+		+		+
R072	1	*Brachybacterium phenoliresistens*		+			+	+	+		+	+	+
R077	4	*Brachybacterium phenoliresistens*					+	+	+		+	+	+
R079	1	*Brachybacterium phenoliresistens*					+	+	+		+	+	+
R081	1	*Streptomyces ambofaciens*						+	+		+		+
R084	1	*Brachybacterium phenoliresistens*					+	+	+	+	+		+
R087	1	*Streptomyces ambofaciens*					+	+	+		+	+	+
R092	2	*Streptomyces ambofaciens*		+		+		+	+				+
R097	1	*Brachybacterium phenoliresistens*	+					+	+				+
R099	6	*Enterococcus faecalis*	+				+	+	+	+	+	+	+
R102	3	*Enterobacter hormaechei*	+			+		+	+	+	+	+	+
R104	2	*Bacillus proteolyticus*		+		+		+	+				+
R105	2	*Streptomyces lavendulocolor*	+					+	+	+			+
R106	1	*Streptomyces finlayi*	+			+		+	+	+	+		+
R112	2	*Streptomyces massasporeus*				+	+	+	+				+
R113	1	*Streptomyces finlayi*	+			+		+	+		+	+	+

R115	4	*Streptomyces ambofaciens*	+	+		+	+		+	+	+
R116	1	*Streptomyces mutabilis*			+	+	+				+
R121	4	*Streptomyces mutabilis*	+	+		+	+				+
R124	3	*Streptomyces mutabilis*	+	+		+	+		+	+	+
R125	1	*Bacillus zanthoxyli*	+	+	+	+	+	+	+	+	+
R126	5	*Streptomyces graminofaciens*	+	+		+	+				+
R131	3	*Streptomyces pulcher*				+	+		+	+	+
R133	3	*Streptomyces pulcher*				+	+	+	+	+	+
R138	13	*Streptomyces macrosporeus*	+			+	+		+	+	+
R140	2	*Bacillus subtilis*		+		+	+	+	+	+	+
R141	2	*Streptomyces mutabilis*				+	+		+	+	+
R142	3	*Streptomyces radiopugnans*	+	+		+			+	+	+
R143	1	*Brachybacterium phenoliresistens*	+		+	+	+		+	+	+
R144	5	*Streptomyces mutabilis*	+			+	+		+	+	+
R147	1	*Microbacterium paludicola*			+	+	+				+
R148	1	*Bacillus subtilis*		+		+	+				+
R150	2	*Streptomyces radiopugnans*	+	+		+	+				+
R151	1	*Brachybacterium phenoliresistens*	+		+	+	+		+	+	+

Supplementary Figures

Supplementary Figure S1. *Stipagrostis pungens* rhizosheath-root system. (**a**) Portion of *S. pungens* rhizosheath-root system (scale bar, 5 mm). (**b**) Root cross section of the rhizosheath-root system visualized at the stereo microscope; RT: root tissue, RS: rhizosheath matrix of root hairs with attached sand grains, CL: visible cortical lysis (scale bar, 500 µm). The thickness of the rhizosheath matrix, measured from the root epidermal surface to the external sand grains, ranged from 0.5 to 0.8 mm; the central root showed a partial lysis of the cortex, as previously observed in other plants during rhizosheath formation [7,8]. (**c**-**e**) representative images of the three components of the rhizosheath-root system: (**c**) central root tissues (without epidermis); (**d**) rhizosheath matrix, starting from epidermis and including all sands grains strongly entrapped by root hairs; (**e**) rhizosphere, all sand grains loosely attached to the rhizosheath and lost in the tube during the sampling procedures (scale bars, 10 mm).

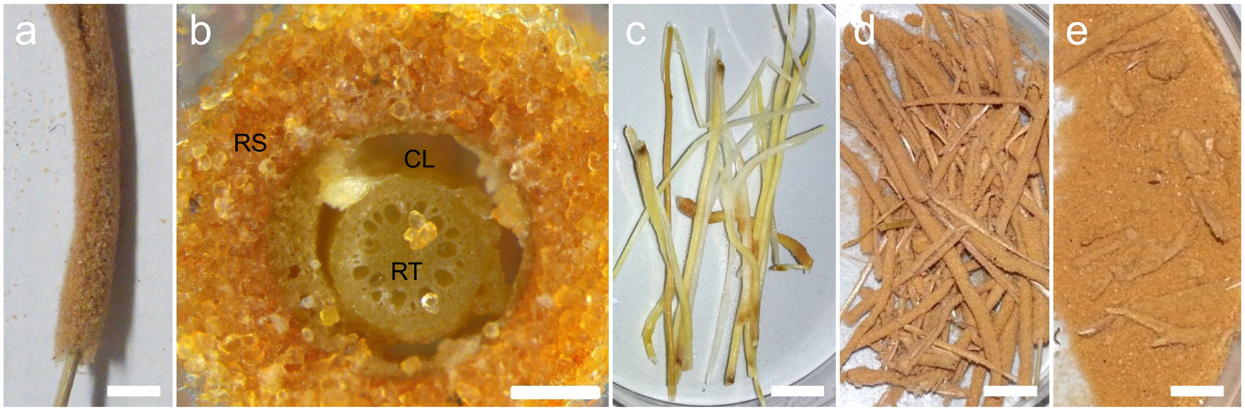

Supplementary Figure S2. Rarefaction curves of bacterial reads obtained by pair-ends MiSeq Illumina sequencing in bulk sand, rhizosheath, rhizosphere, and root tissues; graphs have in the x-axis the number of reads, and in the y-axis the number of KTUs detected (richness). The shape of rarefaction curves indicates that a substantial number of bacterial KTUs were recovered to reach saturation or near saturation.

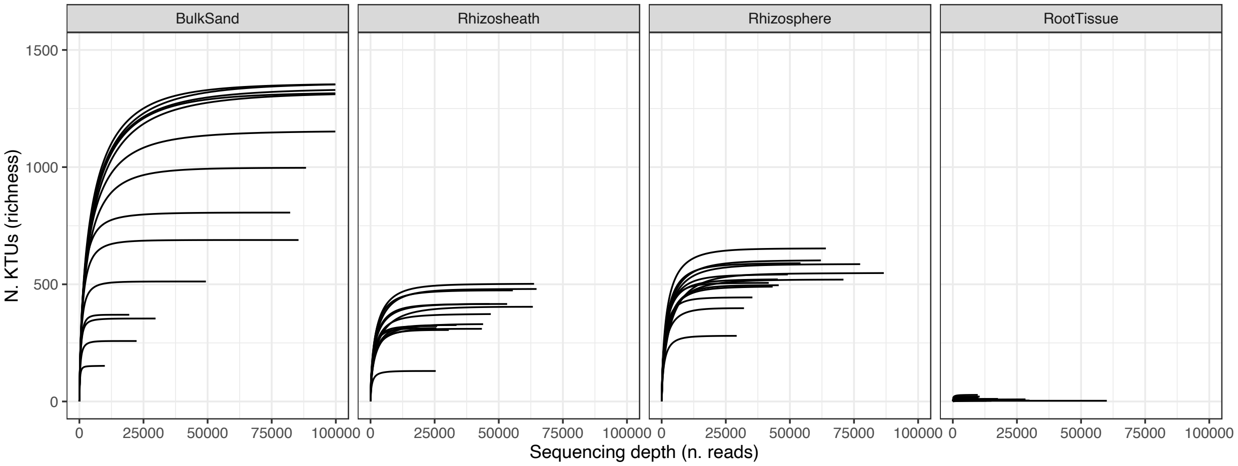

Supplementary Figure S3. Visualization of *Stipagrostis pungens* rhizosheath with scanning electron microscopy (SEM). (**a**) A longitudinal root section of *S. pungens* showing the structure of the rhizosheath enveloping the root (cryo-SEM). (**b**) Root hairs and remnant portion of the mucigels-covering/binding the rhizosheath matrix after the critical point drying process for conventional SEM. (**c**–**n**) Images showing bacteria with different morphologies that colonize the surface of roots, root hairs and sand grains. (**h** and **i**) Flaky/coating materials covering the sand grain surfaces (SEM), and (**m**) mucigels enwrapping bacterial cells (cryo-SEM).

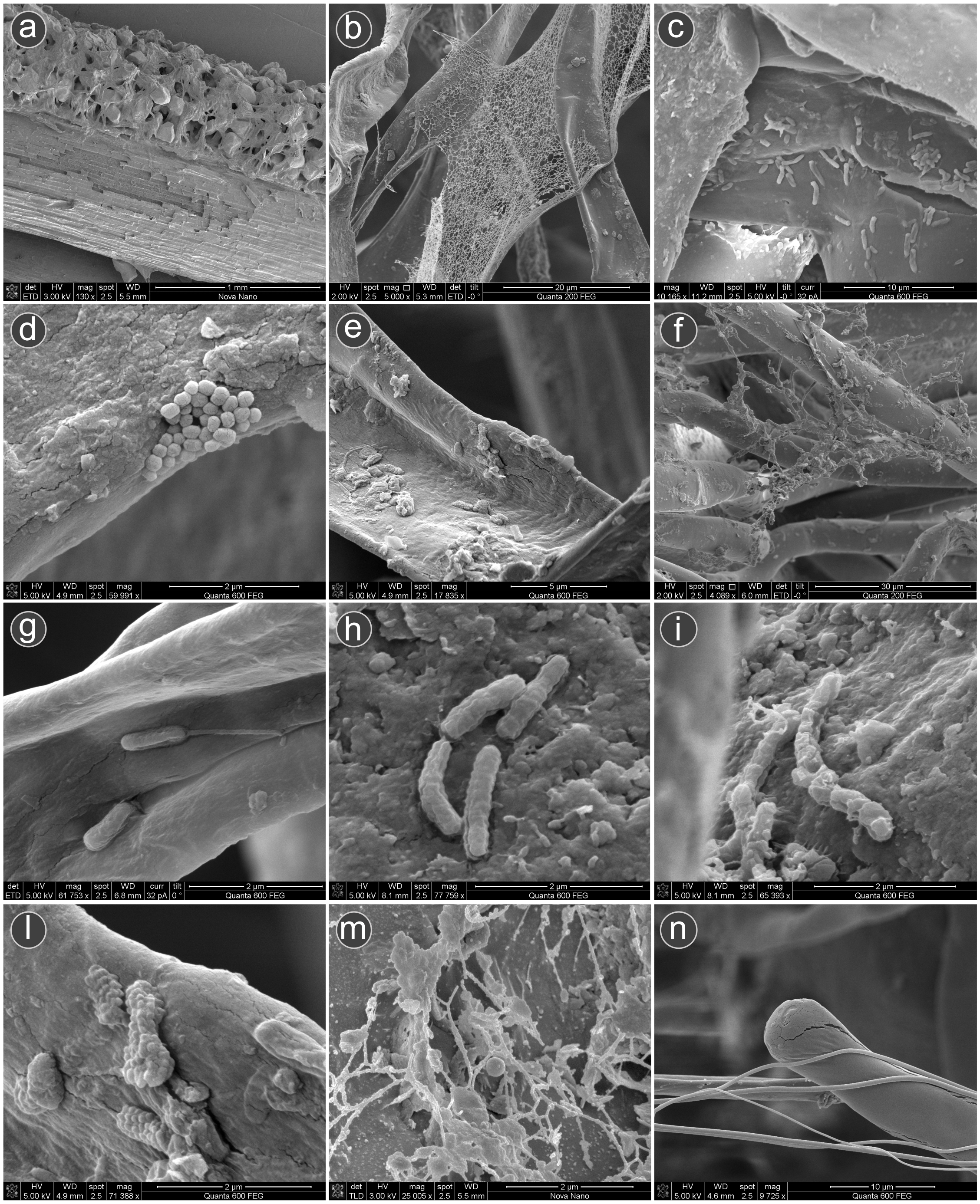

Supplementary Figure S4. SEM images and electron micrographs of the sand grains and root tissue within the rhizosheath of *Stipagrostis pungens* using environmental scanning electron microscopy (ESEM) to reveal chemical composition. (**a**) The aluminium base on which the rhizosheath is mounted is shown as a reference. (**b**–**c**) Sand grain surface analysis showing the dominant contribution of silicon, oxygen, and carbon to sand grain composition; the latter two indicate an organic origin of the mucus sand grains were covered/embedded within. (**d**) Root tissue analysis showing the dominant contribution of carbon and oxygen to root tissue composition. Wt%, weight %; At, atomic %.

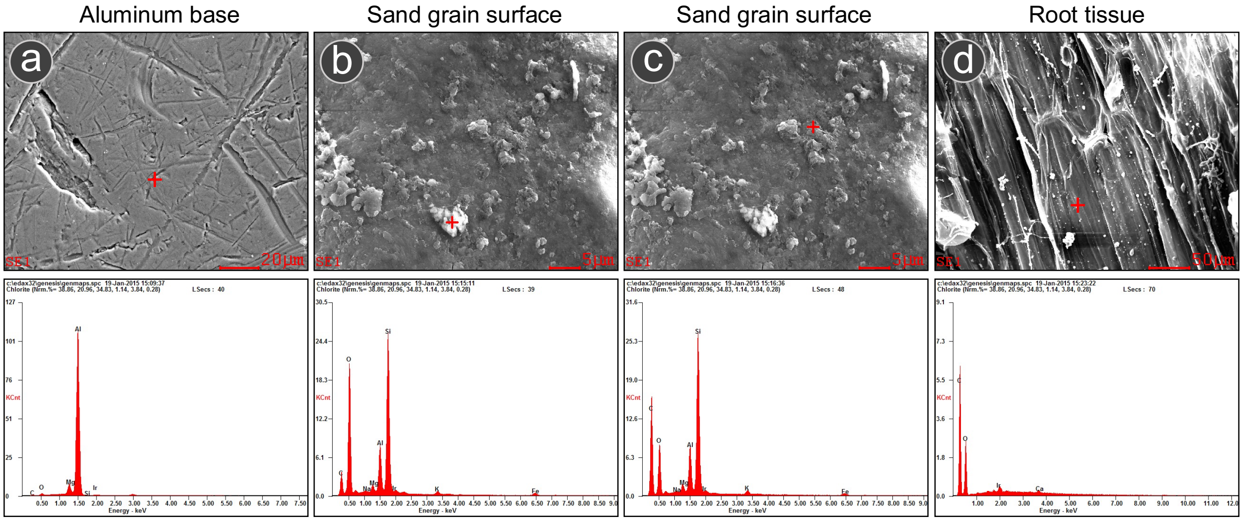

(a) Aluminum base

Element	Wt%	At%
C	03.44	07.37
O	01.84	02.97
Mg	04.76	05.04
Al	88.48	84.39
Si	00.05	00.05
Ir	01.43	00.19

(b) Sand grain surface

Element	Wt%	At%
C	16.91	26.65
O	38.12	45.11
Na	00.28	00.23
Mg	01.14	00.89
Al	08.25	05.79
Si	28.79	19.40
Ir	01.80	00.18
K	01.07	00.52
Fe	03.64	01.23

(c) Sand grain surface

Element	Wt%	At%
C	47.21	63.65
O	17.01	17.21
Na	00.20	00.14
Mg	01.02	00.68
Al	06.42	03.86
Si	22.68	13.07
Ir	01.67	00.14
K	01.16	00.48
Fe	02.64	00.76

(d) Root tissue

Element	Wt%	At%
C	54.74	65.72
O	36.67	33.05
Ir	06.52	00.49
Ca	02.07	00.74

Supplementary Figure S5. Localization of bacteria in rhizosheath-root system by confocal laser-scanning microscopy (CLSM) and fluorescence *in situ* hybridization (FISH). (**a**) Brightfield image of sand grain enwrapped by root hairs in the rhizosheath matrix. (**b**) Rhizosheath samples treated with NONEUB probes; green: autofluorescence of exopolysaccharides. (**c**) Rhizosheath samples treated with EUBMIX probes to visualize the overall bacterial cells; green: autofluorescence of exopolysaccharides, red: all bacteria. (**d**–**f**) Colonization of root hairs and sand grains within rhizosheath samples treated with EUBMIX (all bacteria) and HGC236 (Actinobacteria-specific) probes; green: autofluorescence of exopolysaccharides, orange: Actinobacteria (yellow + red co-signal) red: other bacteria (non-Actinobacteria). Asterisks (*) indicate root hairs and arrows bacterial cells and bacterial cluster. All scale bars, 30 µm.

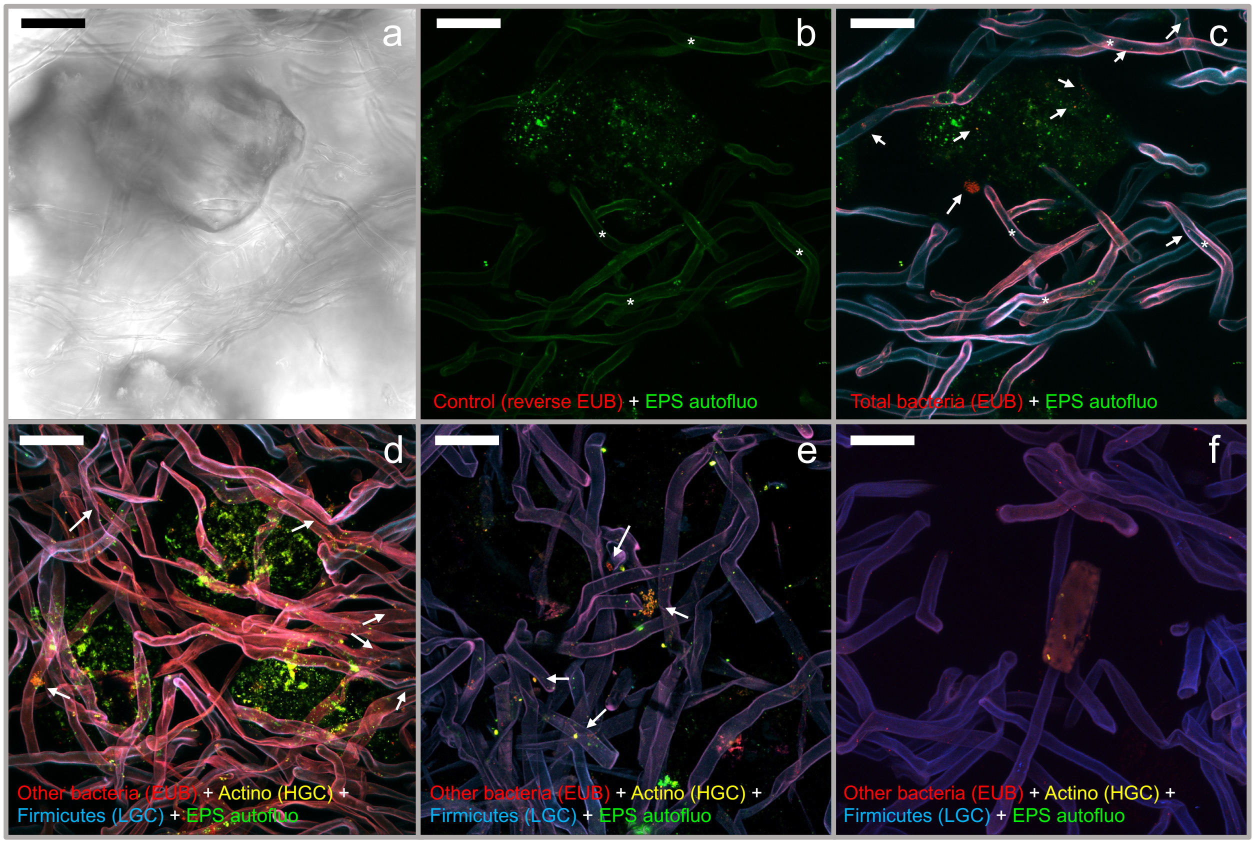

Supplementary Figure S6. Bacterial KTUs distribution across samples. For each line, it is reported the (i) rank KTU distribution in function of KTU relative abundance (%), (ii) the frequency of KTU (expressed as percentage of the n. of samples that have such KTU) in function of KTU relative abundance (%), and (iii) the number of KTUs for each percentage of frequency (bin center, every 5% of frequency). Data are reported for the entire dataset (1st line), and separately for each compartment (2nd line: root tissue; 3rd line: rhizosheath 4th line: rhizosphere; 5th line: bulk sand).

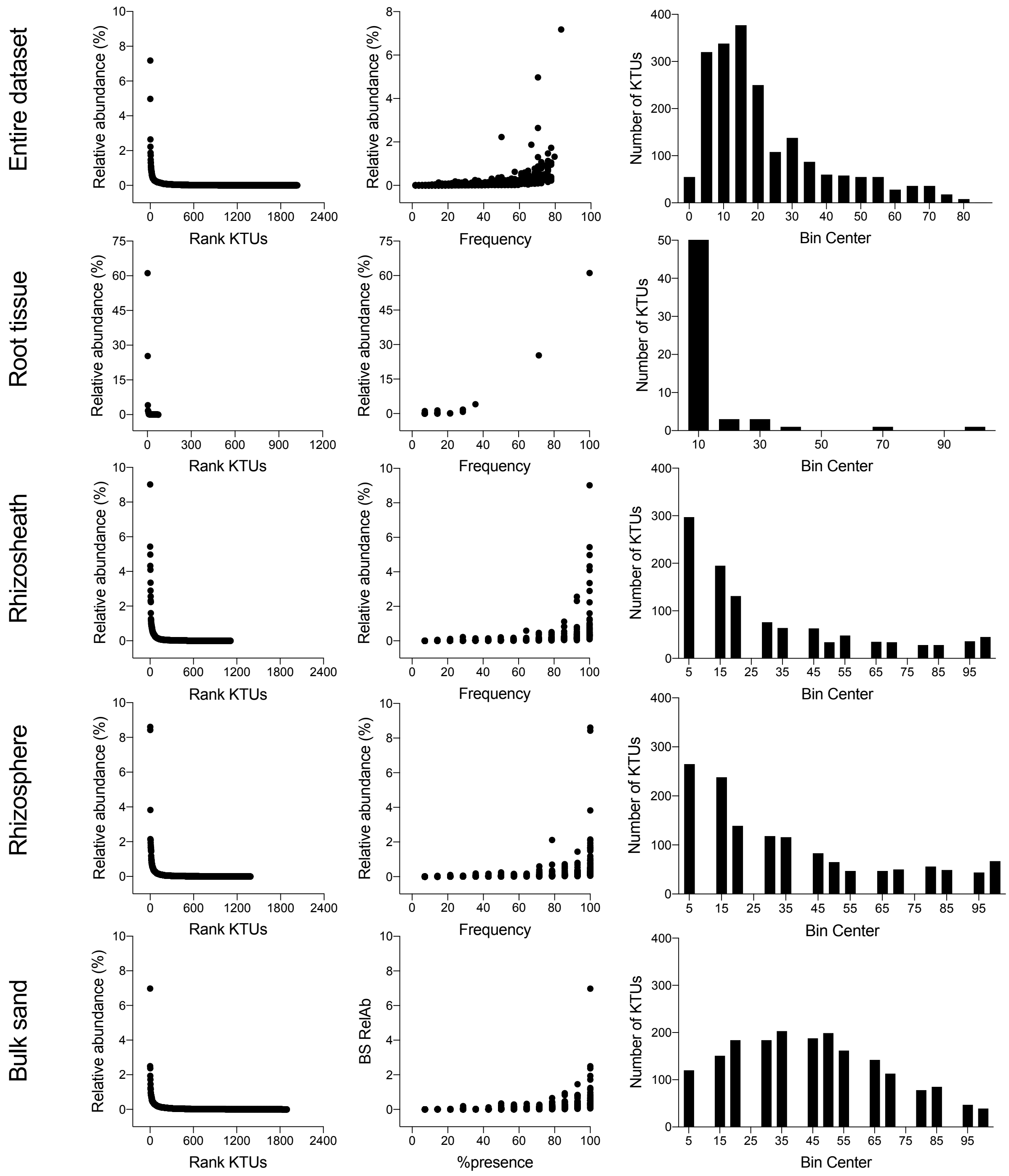

Supplementary Figure S7. Venn diagram shows the distribution of bacterial KTUs across compartment categories (catBS, bulk sand; catRH, rhizosphere, catRS, rhizosheath; catRT, root tissues); values indicate the number of KTUs.

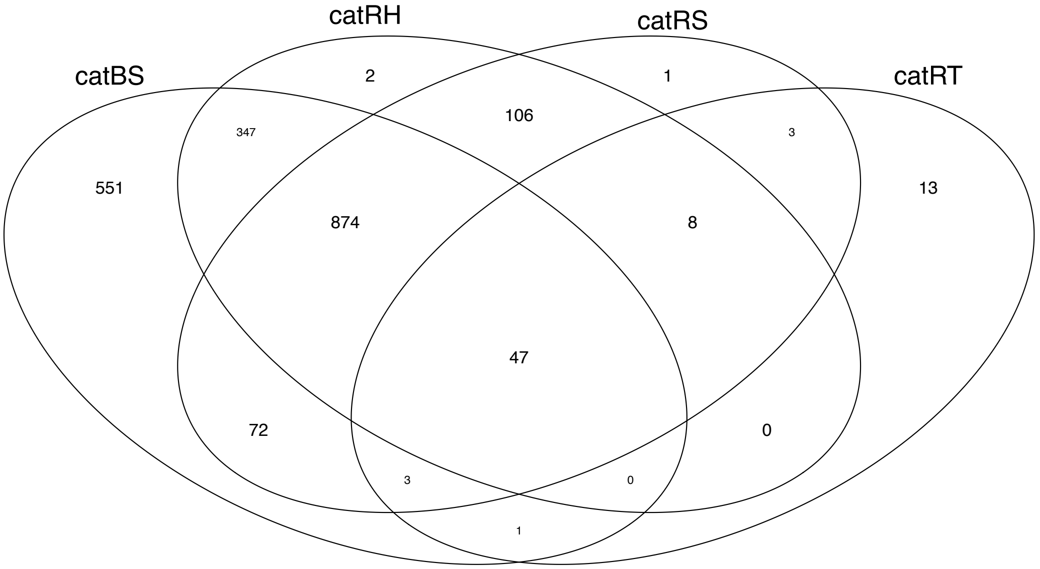

Supplementary Figure S8. Alpha diversity expressed as (a) richness (number of KTUs) and (b) Shannon diversity across the compartment categories (RT, root tissue; RS, rhizosheath; RH, rhizosphere; BS, bulk sand). Lower case letters indicate the results of Tukey' multiple comparison tests (significance, p-value < 0.05).

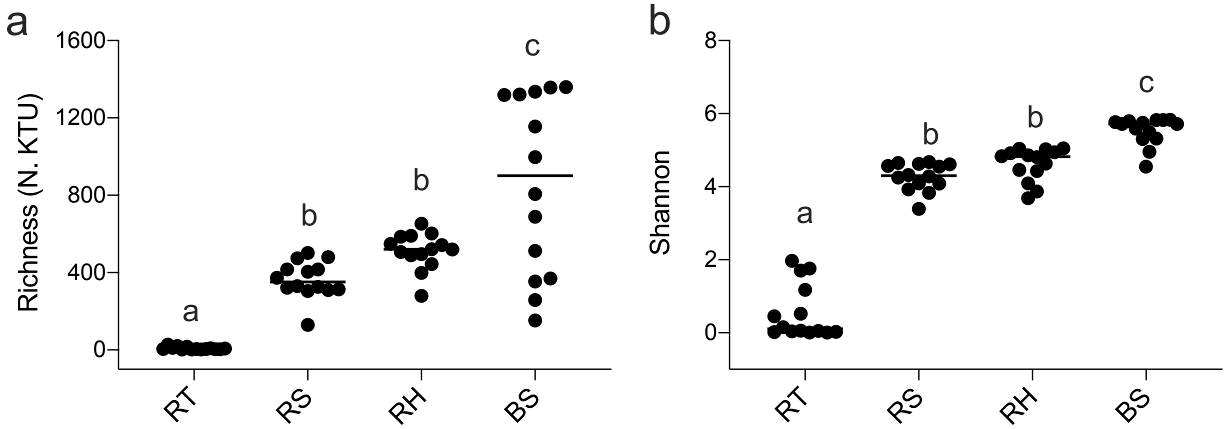

Supplementary Figure S9. Quantification of betadiversity components in bulk sand bacterial communities across seasons (summer and winter). Each point in the ternary plot is determined by a triplet of values from the three betadiversity components: similarity, replacement, and richness difference. In the ternary, large central dots from which the lines start represent the centroid of the points; the lines represent the mean values of the three components).

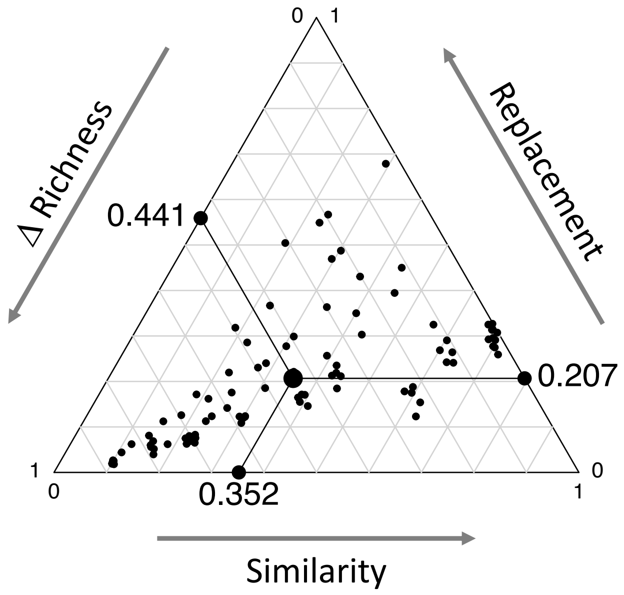

Supplementary Figure S10. Alpha diversity expressed as richness (number of KTUs) and Shannon diversity across the compartments (RT, root tissue; RS, rhizosheath; RH, rhizosphere; BS, bulk sand) in summer (S) and winter (W) seasons. Asterisks (*) indicate the results of comparison tests (significance, p-value < 0.05) among the different compartments in the two seasons.

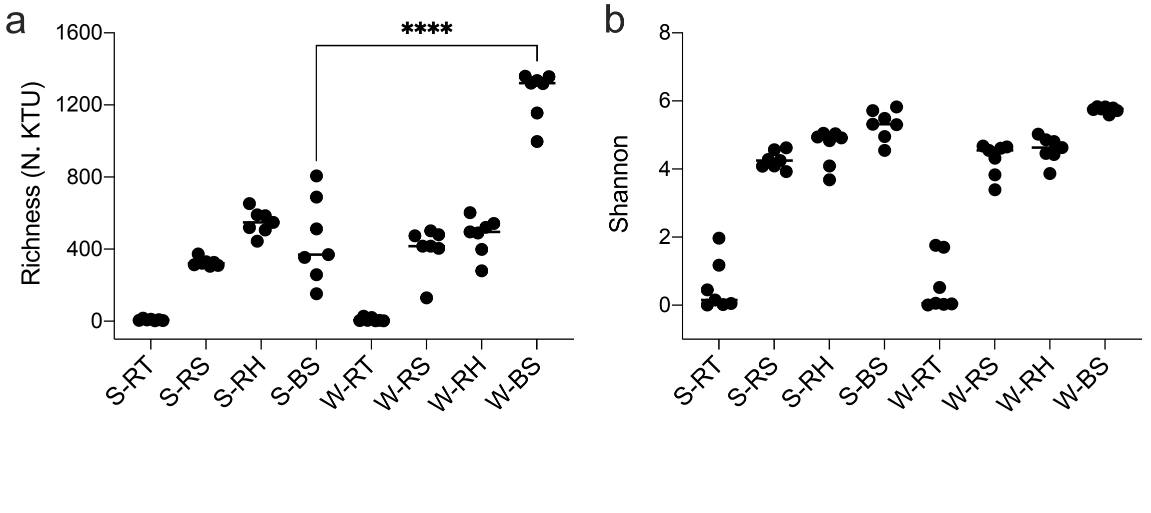

Supplementary Figure S11. Venn diagrams showing the number of KTUs present in summer and/or in winter (catSummer and catWinter, respectively) in each compartment (root tissue, rhizosheath, rhizosphere, and bulk sand); numbers within the Venn diagrams indicate the number of KTUs detected in summer, winter and in both seasons, while the KTUs outside the Venn diagrams indicate the number of KTUs that are present in that specific compartment.

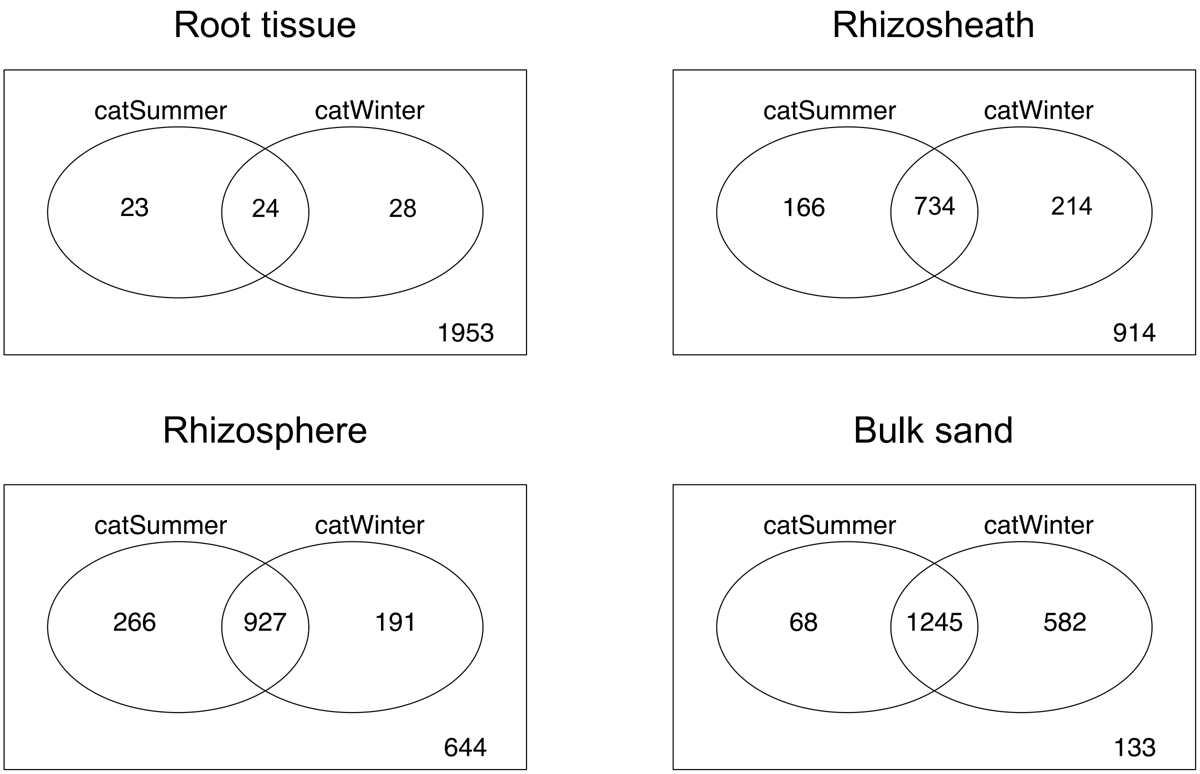

Supplementary Figure S12. Analysis of 2-fold change was performed to evaluate the KTUs that had a significantly (p-value < 0.01) different relative abundance (2-fold change) over summer and winter. The analysis was performed for each compartment: (**a**) root tissue, (**b**) rhizosheath, (**c**) rhizosphere, and (**d**) bulk sand. KTUs enriched in summer and in winter are colored in orange and light blue, respectively; KTUs that did not significantly change in abundance across seasons are shown in black.

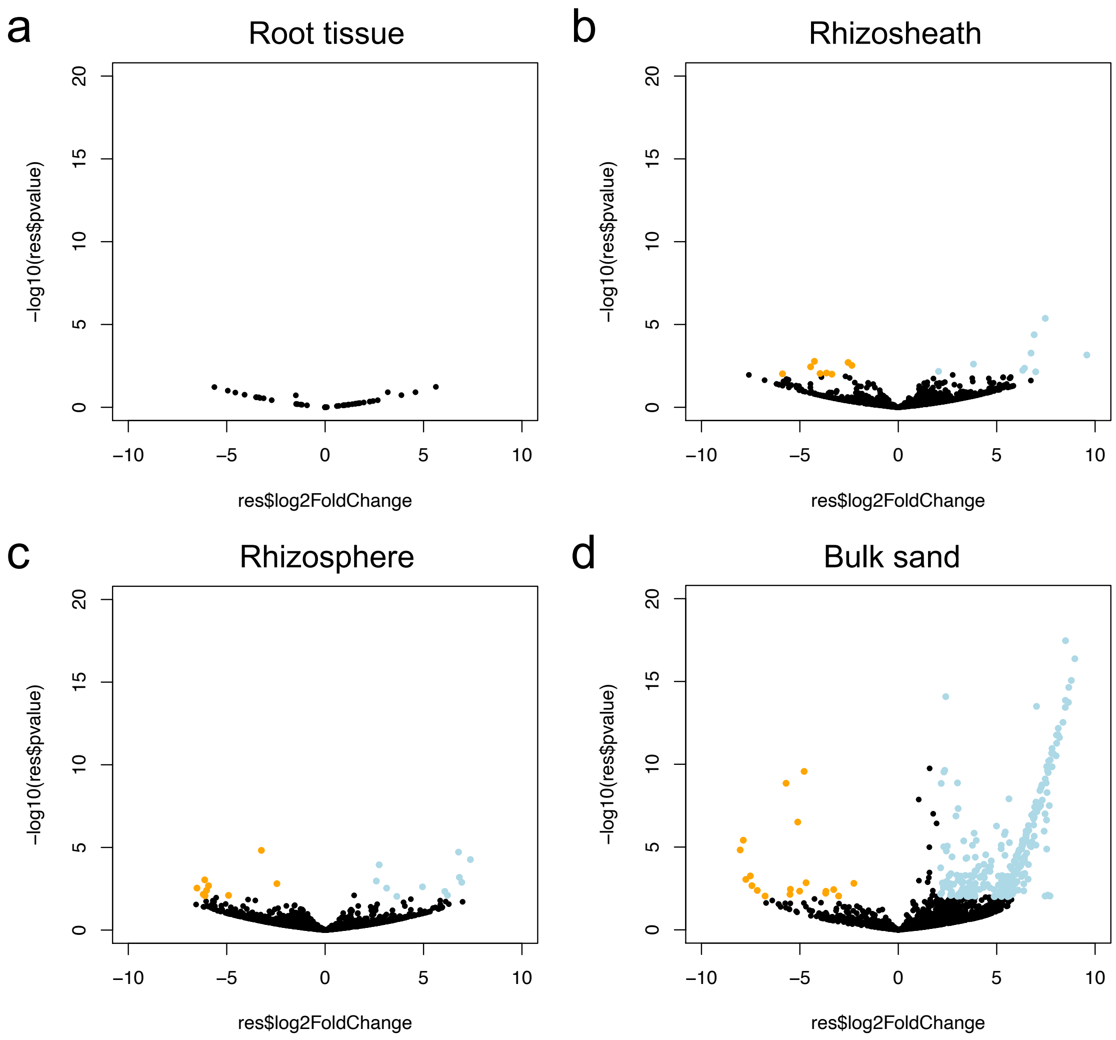

Supplementary Figure S13. Taxonomy of co-occurrence network degrees in bulk sand (BS) and rhizosheath–root system (RS) across two contrasting seasons, summer and winter.

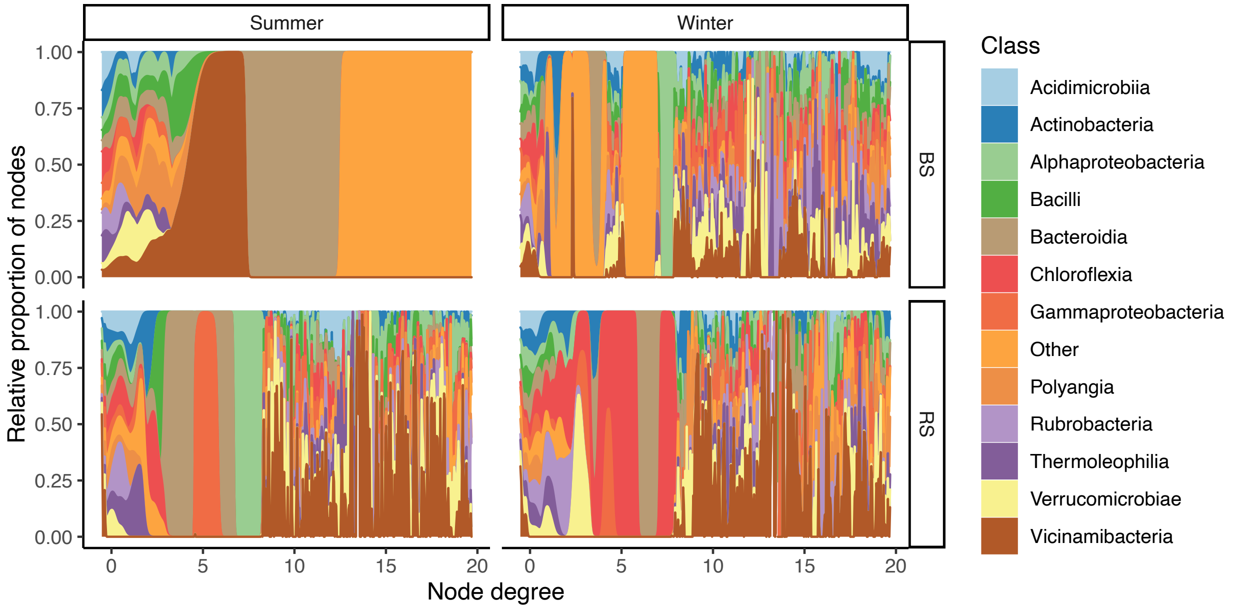

Supplementary Figure S14. Microcosms to evaluate sand wettability *in vitro*. Evaluation of sand weight at 48 h (left panel) and along the entire incubation (0, 24 and 48 h; right panel); values of the three replicates for each treatment are reported and expressed as percentage of send weight respect to the one at the initial time (T_0). Letters indicate results of Tukey's multiple comparisons tests.

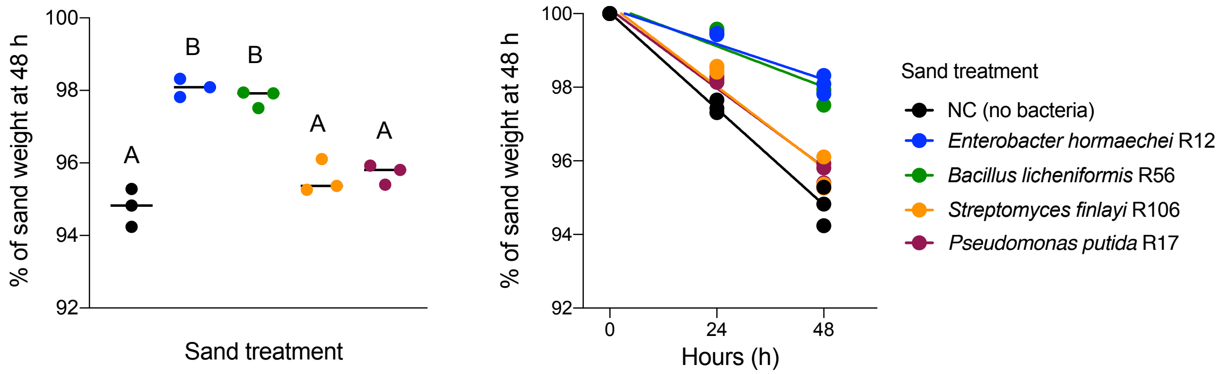

Supplementary Bibliography

1. Amann RI, Krumholz L, Stahl DA. Fluorescent-oligonucleotide probing of whole cells for determinative, phylogenetic, and environmental studies in microbiology. J Bacteriol. Am Soc Microbiol; 1990;172:762–70.
2. Daims H, Brühl A, Amann R, Schleifer K-H, Wagner M. The domain-specific probe EUB338 is insufficient for the detection of all bacteria: Development and evaluation of a more comprehensive probe set. Syst Appl Microbiol. Elsevier; 1999;22:434–44.
3. Neef A. Anwendung der in situ-Einzelzell-identifizierung von bakterien zur populationsanalyse in komplexen mikrobiellen Biozonosen. Ph D thesis Tech Univ Munichen. 1997.
4. Manz W, Amann R, Ludwig W, Wagner M, Schleifer K-H. Phylogenetic oligodeoxynucleotide probes for the major subclasses of proteobacteria: problems and solutions. Syst Appl Microbiol. Elsevier; 1992;15:593–600.
5. Erhart R, Bradford D, Seviour RJ, Amann R, Blackall LL. Development and use of fluorescent in situ hybridization probes for the detection and identification of "Microthrix parvicella" in activated sludge. Syst Appl Microbiol. Elsevier; 1997;20:310–8.
6. Wallner G, Amann R, Beisker W. Optimizing fluorescent in situ hybridization with rRNA-targeted oligonucleotide probes for flow cytometric identification of microorganisms. Cytometry. Wiley Online Library; 1993;14:136–43.
7. Wullstein LH, Bruening ML, Bollen WB. Nitrogen fixation associated with sand grain root sheaths (rhizosheaths) of certain xeric grasses. Physiol Plant. 1979;46:1–4.
8. Wullstein LHH, Pratt SAA. Scanning electron microscopy of rhizosheaths of Oryzopsis hymenoides. Am J Bot. 1981;68:408–19.
